# Toward Optical Quality Polylactide Using Fatty Acid Amides as Clarifiers

**DOI:** 10.1021/acs.biomac.5c01123

**Published:** 2025-08-01

**Authors:** Matthias Balthasar Kesting, Sebastian Hochstädt, Eric Terbrack, Anna Maria Ruder, Bernd Ahrens, Walter Chassé, Christian Thomas, Stefan Schweizer, Michael Ryan Hansen, Jörg Meyer, Gunnar Seide

**Affiliations:** † Department Lippstadt 1, 233533 Hamm-Lippstadt University of Applied Sciences, Marker-Allee 76-78, 59063 Hamm, Germany; ‡ Aachen-Maastricht Institute for Biobased Materials (AMIBM), 5211 Maastricht University, Bright-lands Chemelot Campus, Urmonderbaan 22, 6167 RD Geleen, The Netherlands; § Institute of Physical Chemistry, 9185 University of Münster, Corrensstraße 28/30, 48149 Münster, Germany; ∥ Faculty of Electrical Engineering, South Westphalia University of Applied Sciences, Lübecker Ring 2, 59494 Soest, Germany; ⊥ Fraunhofer Application Center for Inorganic Phosphors, Branch Lab of Fraunhofer Institute for Microstructure of Materials and Systems IMWS, Lübecker Ring 2, 59494 Soest, Germany

## Abstract

Polylactide (PLA) represents a sustainable alternative to common optical polymers in lighting applications. However, its application temperature is currently limited, since the material crystallizes and becomes cloudy when exposed to temperatures above 55–65 °C. Here, *N*,*N*′-ethylenebis­(stearamide) and *N*,*N*′-ethylenebis­(12-hydroxystearamide) are applied as clarifiers, and their effects are known for foils. This approach is extended to bulky materials (*d* = 1.5 mm). Optical characterization was complemented by microscopy, differential scanning calorimetry (DSC), and X-ray diffraction (XRD). Additionally, NMR was applied to understand the effects of the fatty acid amides on a molecular level. All methods confirm the nucleating effects of both additives, while the crystallinity and crystal structure remain unchanged. A controlled crystallization at lower temperatures is discovered to promote the effect of the clarifiers by further reducing crystallite size. Finally, the crystallized samples are exposed to 80 °C and found to maintain high optical transmission. A tempering step at low temperatures is suggested to maximize clarifying effects.

## Graphical abstract

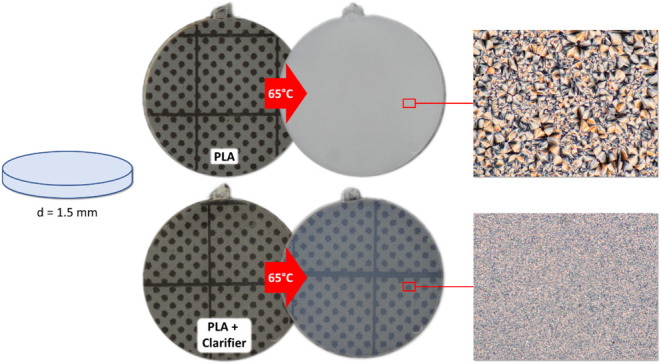

## 1 Introduction

In 2017, lighting applications were estimated to consume approximately 16.5% of the global energy production.^1,2^ Relative to 2010, this represents a drop by 2.5% of this share. Simultaneously, the penetration rate by sales of light emitting diodes (LEDs) increased from almost 0% in 2010 to 26.3% in 2017.^2^ A market penetration rate of 75.8% for LEDs is projected by 2025.^2^ LED light sources offer key advantages, notably high efficiency and the ability to achieve high luminous flux.^3,4^ Due to relatively low heat dissipation, a minimum temperature stability of only 85 °C is required for optical parts in LED applications, which allows the use of polymers that offer cost-effective production and ease of processing.^5^ However, common optical polymers such as polycarbonate (PC) and poly methyl methacrylate (PMMA) are fossil based and hardly degradable in the environment.^6,7^ Their sustainability is therefore questionable when considering the widely discussed plastic problem.^8–10^

Optical systems place challenging demands on the materials used. These include a direct transmission >90%, a refractive index of ∼1.5 and an Abbe number >30 as well as mechanical properties including a Young’s Modulus of ∼2500 MPa, a tensile strength >30 MPa, a heat deflection temperature (1.8 MPa) > 85 °C, and a Vicat Penetration Temperature (50 N) > 80 °C.^5^ Fortunately, the Bioplastic polylactide (PLA) does show the required optical properties in the amorphous state,^5,11^ while most the mechanical requirements are fulfilled in semicrystalline PLA.^12^ Considering photothermal degradation PLA was proven to surpass the stability of the highly used PC.^3,13,14^ However, conventional semicrystalline PLA is a volume scatterer and has a directed transmission of 0%.^15^ This is due to crystallites with spherulitic morphology: When the crystals reach a certain size (around 200 nm) they scatter light in accordance with Mie scattering at phase boundaries between amorphous and crystalline domains. Because PLA crystallizes above its glass transition temperature (*T*_g_) (approximately 55–65 °C), this currently limits the application temperature of transparent PLA.^16–19^

To attain transparent and semicrystalline polymers, a variety of approaches may be considered, including the reduction of crystallite size as well as introduction of highly oriented crystal structures.^16,20,21^ This is usually achieved by the addition of specific nucleating agents. Such additives are also referred to as clarifiers, since they reduce light scattering and enhance clarity.^22^ In polypropylene (PP), clarifiers are well established. In this context, the effectiveness of clarifiers is associated with their self-organization into nanofibrillar networks upon cooling. While the clarifiers are dissolved in the molten PP matrix, they segregate during cooling and crystallize prior to the PP.^23^ For the formation of nanofibrillar networks, it is crucial that the additives possess an amphiphilic structure, enabling self-assembly into 3D networks due to their distinct hydrophilic and hydrophobic domains.^22^ The formed network has been shown to provide surfaces that are both physically and chemically conducive to heterocrystallization, thereby substantially increasing the nucleation density and consequently leading to a reduction in crystal size.^24–26^ Considering PLA, clarifiers have been studied for thin foils only. Two fatty acid amides, namely *N*,*N*-ethylenebis­(stearamide) (EBS) and *N*,*N*-ethylenebis­(12-hydroxystearamide) (EBHS), were used in this regard. Samples with a thickness of 250–500 μm and a low weight percentage (wt %) of either EBS or EBHS were shown to retain high transparency after temperature treatments with up to 100 °C. The samples were confirmed to be in a semicrystalline state by different methods. Furthermore, clues were found suggesting EBS and EBHS to self-organize into a nanofibrillar networks.^27–32^

For optical applications, these results have to be transferred to bulky materials since optical components are usually much thicker than foils (usually thickness is in the range of a few millimeters). For a given degree of scattering the clarity (i.e., direct transmission) decreases exponentially with increasing thickness.^33^ Therefore, the crystallization induced by EBS and EBHS requires precise tuning, with careful adjustments to the materials’ requirements and with regard to the external influences that affect it. Once crystallized, it is assumed, that semicrystalline PLA has a constant number of crystallites and therefore invariable optical transmission, also at higher temperatures. It is described by Schmidt et al. how the crystal size and number in a fully crystallized material is invariant during heating even when undergoing α′/α-transition.^34^

For the first time, this study demonstrates the application of EBS and EBHS as clarifiers in PLA samples with thicknesses relevant for optical applications. To this end, the crystallization in the material is finely tuned by the introduction of a heat treatment at particularly low temperatures just above *T*_g_, followed by in depth characterization of the crystalline morphology. The study is complemented by the characterization of the crystallization kinetics of the compounds via dedicated low- and high-field NMR techniques. It is demonstrated that the used clarifiers influence the crystallization process and molecular mobility of the polymer chains. By this, a significant contribution to the understanding of the mode of action of these additives is made, which will enable enhancing the optical performance of PLA in lighting applications. Our approach is novel considering (a) relevant sample thickness and (b) the characterization of crystallization on a molecular level by NMR. Both provide access to the use of PLA as sustainable optical material for lighting applications.

## 2 Experimental Section

### 2.1 Materials

PLA Luminy L130 (l-isomer content ≥ 99%) with a molecular weight of approximately 115 kg/mol (own measurement with Prominence Liquid Chromatograph, (Shimadzu, Kyoto, Japan) calibrated with PSS-mmkitr1 standard) was selected and kindly provided by TotalEnergies Corbion (Gorichem, Netherlands). Other chemicals used for sample preparation were chloroform (purity ≥ 99.8%, stabilized with amylene) from Sigma-Aldrich (St. Louis, Missouri), 2-propanol (purity of 99.9%) from Höfer Chemie (Kleinbittersdorf, Germany), *N*,*N*′-ethylenebis­(stearamide) (EBS) beads <840 μm (purity not specified) from Sigma-Aldrich (St. Louis, Missouri), *N*,*N*′-ethylenebis­(12-hydroxystearamide) (EBHS) powder, purity: 90%, from Alfa Chemistry (Ronkonkoma, New York) and grade 424 paper filters from VWR (Radnor, Pennsylvania). For temperature treatment and drying a VO500 vacuum oven with PMP500 vacuum pump from memmert (Schwabach, Germany) was used.

### 2.2 Sample Preparation

A powder mixing process with subsequent injection molding was used to prepare the sample specimens. Before processing, the materials were dried at 60 °C for at least 24 h in a vacuum oven at 50 mbar. EBS and EBHS were purified by dispersion in chloroform with subsequent filtration. PLA was ground by means of a CryoMill from Retsch (Haan, Germany) for five times 5 min at 30 Hz with 30 s intermediate cooling (liquid nitrogen) and 2 min precooling (liquid nitrogen) at 5 Hz, respectively. The powders obtained were sieved by an AS 200 basic sieve tower with a 500 μm sieve from Retsch (Haan, Germany) and mixed at 2300 rpm for 2 min in a DAC 150.1 FVZ-K SpeedMixer from Hauschild (Hamm, Germany) in appropriate mass ratios (see Table 1). The samples were injection molded from the compound powder using a HAAKE Minijet Pro injection molding apparatus from Thermo Fisher Scientific (Waltham, Massachusetts) at 195 °C cylinder temperature and 23 °C mold temperature at 600 bar injection pressure for 5 s and 400 bar after pressure for 3 s. Their geometry was disk-shaped with a diameter of 20 mm and a thickness of 1.5 mm. Sample names and composition are listed in Table 1. These labels were suffixed with applied tempering temperature in °C where appropriate. Pictures of the samples were taken with an EOS 1300D with EF-S 18–55 mm f4–5.6 lens, both from Canon (Tokyo, Japan).

**Table 1:** Sample Names and Composition Considering Their Mass Ratios

sample	PLA (%)	EBS (%)	EBHS (%)
PLA	100.0	0.0	0.0
PLAEBS	99.5	0.5	0.0
PLAEBHS	99.5	0.0	0.5

### 2.3 Temperature Treatment

Temperature is one of the major influencing factors considering spherulitic growth rates and nucleation densities in PLA crystallization.^35^ To follow previous approaches, the PLA compounds were first treated at 100 °C. Subsequently, temperatures were lowered to 75 °C and finally 65 °C to promote nucleation over crystal growth. For every temperature a total of five disc-shaped samples was tempered per compound. The samples were placed on a thin aluminum plate and transferred into the oven at environmental pressure. During each temper procedure the samples were analyzed at defined time intervals with ultraviolet-visible (UV–Vis) and Fourier transform infrared (FTIR) spectroscopy. When the direct light transmission spectra reached a stable value, crystallization was considered complete. The temperature treatment was stopped and one sample of each compound and temperature respectively was selected for analysis with destructive methods, i.e., differential scanning calorimetry (DSC) and X-ray diffraction (XRD).

### 2.4 Direct Light Transmission

The samples were characterized considering their transmission properties with respect to electromagnetic radiation in the wavelength range from 200 to 800 nm using a UV–Vis spectrometer UV-2600 from Shimadzu (Kyoto, Japan) before, during and after temperature treatment. The characterizations were carried out with a spectral resolution of 2 nm. A baseline was measured preliminary to any characterizations.

For samples *i* = 1 to *i* = *n* the individual transmission spectra *T*_*i*_ (λ) were received. The average transmission spectrum *T*(λ) was calculated by taking the average of all *n* measurements at wavelength λ. This is expressed by the following eq 1.

1 $$T\left(\lambda \right)=\frac{1}{n}\sum_{i=1}^{n}T_{i}\left(\lambda \right)$$

The average direct light transmission *T* in the visible range (i.e., from 360 to 780 nm) was calculated by eq 2.

2 $$T=\frac{1}{780-359}\cdot \sum_{\lambda =360}^{780}T\left(\lambda \right)$$

### 2.5 Diffuse Reflectance

The diffuse reflectance was measured using the ISR2600 integrating sphere attachment for the UV–Vis spectrometer by Shimadzu (Kyoto, Japan), in accordance with the manufacturers’ guidelines. Spectra were measured with a spectral resolution of 2 nm in the wavelength region from 200 to 800 nm. Three samples per compound and temper temperature were analyzed, except for PLA_100 where one sample broke during the investigation leaving two samples for analysis. The mean reflectance spectra *R*(λ) were calculated in accordance with eq 1.

### 2.6 Nuclear Magnetic Resonance Spectroscopy

As a supplement to the commonly used analytical methods to investigate the structure, bulk crystallinity, and crystallization kinetics of semicrystalline polymers, low-field nuclear magnetic resonance (NMR) presents unique advantages. Combining the nondestructive nature of NMR with its inherent flexibility, low-field NMR allows for cost-effective and straightforward in situ experiments that can be implemented with minimal sample preparation. This method enables continuous observation of crystallization processes directly within processing or quality control environments, making it particularly suitable for real-time analyses. Therefore, low field NMR is applied to get insights into the mode of action of EBS and EBHS on the crystallization behavior of PLA on a molecular scale. In order to get information about the structure of growing crystallites, additional high-field NMR measurements were carried out and analyzed.

#### 2.6.1 NMR Equipment and Experimental Details

The thermal history of all samples was erased by heating to 200 °C for 30 min under argon atmosphere followed by cooling to ambient temperature at a rate of 10 K/min prior to the solid-state NMR measurements. Solid-state ^13^C­{^1^H} MAS NMR experiments were conducted on a Bruker Avance III 300 spectrometer (υ_L_(^1^H) = 300.16 MHz, 7.05 T) using a Bruker 4.0 mm H/X WVT double-resonance probe. The samples were contained in 4.0 mm ZrO_2_ rotors, sealed with Vespel top-caps. Spectra were acquired at a MAS frequency of 10.0 kHz, with variable temperatures managed by a Bruker VT 3000 temperature control unit. Temperature calibration was performed using ^207^Pb, referenced to the absolute chemical shift of ethylene glycol.^36^ ^13^C chemical shifts and radio frequency (rf) field strengths were calibrated using adamantane (δ­(^13^C) = 38.5 ppm/29.5 ppm) as a secondary reference,^37^ while calibration of the magic angle was performed using ^23^NaNO_3_. Typical parameters for the CP experiments included ^1^H 90° pulses of 4 μs (υ_rf_ = 62.5 kHz) and ^13^C 90° pulses of 5 μs (υ_rf_ = 62.5 kHz). A cross-polarization (CP) contact time of 4.0 ms with a ramp from 70 to 100% on the ^13^C channel was used. Proton decoupling was employed using the SW_f_TPPM scheme with υ_rf_ = 62.5 kHz coupling strength during CP acquisition.^38^ All experiments were carried out using a recycle delay of 6 and 1200 scans. Processing, data analysis, and plotting were carried out using the Bruker Topspin 4.1.1 software and OriginPro2020b.

All ^1^H NMR experiments were conducted using a Bruker minispec mq20 (Bruker BioSpin GmbH, Rheinstetten, Germany) operating at *B*_0_ = 0.5 T and ω_0_/2π = 19.65 MHz for ^1^H. Temperature regulation was maintained with a Bruker VT 3000 unit, using pressurized nitrogen at a flow rate of 1200 l/h. The NMR probe specifications included a dead time of ∼8 μs, pulse durations of 2.6 μs for 90° pulses, and 5.1 μs for 180° pulses at 0 dB attenuation, with a full-width half-maximum (fwhm) bandwidth of ∼500 kHz.

For crystallization experiments, a combined magic-sandwich echo (MSE) decay (initial τ = 25 μs), was followed by a Carr–Purcell–Meiboom–Gill (CPMG) echo train (τ_CPMG_ = 25 μs) with phase cycling.^39,40^ A total of 1 cm^3^ PLA pellets were loaded into 10 mm diameter borosilicate NMR tubes, purged with argon, evacuated, and sealed to minimize convection and oxidative degradation upon tempering.

#### 2.6.2 NMR Theory

##### 2.6.2.1 Longitudinal Relaxation and BPP Model

Analysis of local molecular structures and dynamics can be performed by observing the longitudinal *T*_1_ relaxation time via temperature-dependent ^1^H NMR measurements. In this work, *T*_1_ relaxation times are determined in a temperature range from 25 to 200 °C at a Larmor frequency of ω_0_ = 19.65 MHz. The BPP theory developed by Bloembergen, Purcell and Pound describes the fluctuations of the dipolar interaction induced by random isotropic motion and is used here to fit the *T*_1_ data.^41,42^ This model considers a single, random and isotropic motion characterized by a single correlation time, τ_c_ and holds under the Redfield limit (τ_c_ ≪ *T*_1_, *T*_2_).By using the BPP model, correlation times τ_c_ of the observed motional modes of the individual samples are determined and compared with each other.^43^

##### 2.6.2.2 CPMG Multiple Echo Sequence: Crystallization Kinetics via Benchtop NMR

The fraction of rigid protons is anticipated to diminish in amorphous polymers as temperatures rise above the *T*_g_, leading to enhanced molecular mobility. Specifically, for amorphous polymers, a significant increase in mobility is expected around temperatures of approximately *T*_g_ + 50 °C, facilitating the preaveraging of dipolar coupling. For semicrystalline polymers, the rigidity in the crystalline regions at temperatures above *T*_g_ is maintained, which means that the rigid fraction χ­(*T*) in this region serves as a measure of crystallinity. It is assumed that the plateau in which this is valid starts at approximately *T*_g_ + 100 °C and ends when the polymer crystals begin to melt. The presence of gradual transitions between phases of differing mobilities necessitates careful categorization in determining whether a proton should be classified as “crystalline”, “interfacial”, or “amorphous”. This classification ambiguity can lead to discrepancies in results, contributing to the variations in absolute crystallinity when compared to other characterization techniques like DSC.^44,45^

Here, the CPMG multiple echo sequence (according to Carr, Purcell, Meiboom and Gill) is used to distinguish between crystalline and amorphous regions containing protons.^40^ The concept of this method is based on the fact that only highly mobile protons of the amorphous regions are detected by the CPMG sequence,^46^ i.e., these protons are selected based on the refocusing properties of the 180° pulses and relaxation time before the first 180 °CPMG pulse τ_initial_. To achieve a quantitative detection of all mobile protons, we employed a short MSE recording interval (τ_initial_ = 25 μs) in which the signal decay of only the rigid protons takes place. Accordingly, protons with intermediate mobility will be recorded as a fast decaying component in the CPMG block (see Figure 10b). The CPMG intensities *I*_CPMG_ (*t*_exp_) are back extrapolated to *t*_NMR_ = 0 ms and then compared with the back extrapolated CPMG intensity at *t*_exp_ = 0 min, corresponding to a supercooled melt state of the sample at *T*_cryst_. In this way, measurement of crystal fraction is independent of the MSE efficiency.

3 $$X_{c,\mathrm{indirect}}\left(t_{\exp}\right)=1-\frac{I_{\mathrm{CPMG}}\left(t_{\exp}\right)}{I_{\mathrm{CPMG}}\left(t_{\exp}=0\right)}$$

Fitting uncertainties, systematic errors in the fitting process, and variations in NMR sensitivity can be addressed by incorporating a correction factor into eq 3. However, given that the primary objective of these experiments is to compare crystallization kinetics, the analysis is focused on relative crystallinities.^45^

### 2.7 Differential Scanning Calorimetry

To assess the sample’s aggregate condition considering crystallinity, anisothermal differential scanning calorimetry (DSC) measurements were conducted with a DSC 1 from Mettler-Toledo (Columbus, Ohio). (10 ± 0.5) mg sample were placed in an aluminum crucible with a volume of 40 μL (ME-00026763, Mettler Toledo, Gießen, Germany) and heated to 200 °C at a constant rate of 10 K/min. To identify *T*_g_, the inflection point was determined. The initial degree of crystallinity *X*_c_ can be calculated. It is defined by the difference of melting enthalpy Δ*H*_m_ and enthalpy of crystallization happening during the measurement Δ*H*_c_, divided by the reference melting enthalpy for PLLA crystals having an infinite size Δ*H*_m_^0^ = 93.6 J/g.^47^ As this is given in percent, the fraction is nominalized by 100%, resulting in the following expression eq 4.

4 $$X_{c}=100\%\cdot \frac{\Delta H_{m}-\Delta H_{c}}{\Delta H_{m}^{0}}$$

Δ*H*_c_ and Δ*H*_m_ are given by the areas of the crystallization and melting peak, respectively in the DSC thermograms. A baseline was defined from the first onset of crystallization to the last trace of melting.

All DSC measurements were carried out under 50 mL/min nitrogen flow and the results normalized by the sample weight.

### 2.8 Fourier Transform Infrared Spectroscopy

Fourier-transform infrared (FTIR) spectroscopy was conducted using a Nicolet iS50 with Smart iTR attenuated total reflectance (ATR) accessory using a germanium (Ge) crystal (Thermo Fisher Scientific, Waltham, Massachusetts) to investigate the changes in crystallinity of the PLA samples during thermal treatment. The detector used was DTGS KBr (deuterated triglycine sulfate with potassium bromide window), operating with a signal amplification factor of 2.0 and at a scan speed of 0.4747 cm/s. Spectra were collected in the wavenumber range of 4000 to 800 cm^–1^, with a spectral resolution of 8 cm^–1^. Each spectrum was averaged over ten scans to improve the signal-to-noise ratio.

### 2.9 X-ray Diffraction

X-ray diffractograms were measured using an Empyrean II X-ray diffractometer (XRD) by Malvern Panalytical (Malvern, U.K.) with copper K-Alpha source (λ = 1.5406 Å) and recorded over a 2θ range of 10 to 30°. The X-ray tube was operated at a voltage of 40 kV and a current of 40 mA to provide adequate intensity for diffraction measurements. Data acquisition was performed in continuous scanning mode, with a step size of 0.02° and a counting time of 1 s per step to ensure a high signal-to-noise ratio and accurate peak identification. The obtained diffraction patterns were analyzed to verify the crystallinity of the samples.

In situ temperature treatment was conducted with an HTK 1200N high temperature chamber by Anton Paar (Ostfildern-Scharnhausen, Germany). The samples were placed in the setup at 30 °C and initially measured to confirm their amorphous state. Subsequently, the chamber was heated to 60 °C within 3 min and the samples were measured again. One measurement cycle took a total of approximately 25 min. After additional 5 min of waiting, the third measurement was conducted to achieve a temporal resolution of 30 min. This was repeated for the first ten plus the initial measurements. Thereafter the measurement increment was set to 1 h. The experiment was completed after a total of 64 h.

### 2.10 Polarized Light Microscopy

The formation of crystallites was examined by polarized optical microscopy (POM) using an Axio Scope.A1 microscope equipped with EC Epiplan 10*x*/0.35 HD Lens, two polarizers (Polarizer Slider Rotatable and Polarizer D, 90° rotatable with color filter carrier) all by Zeiss (Oberkochen, Germany) and a 4000D digital camera from Canon (Tokyo, Japan) attached via a C-Mount by LMscope (Graz, Austria). A HCS622GXY hot/cold stage from Instec (Boulder, Colorado) allowed in situ temperature treatment of the materials. The temperature of the heating stage was controlled by a mk2000 controller with an attached LN2-P2A running on water, both manufactured by Instec (Boulder, Colorado). A 10 mm by 10 mm coverslip was cleaned with 2-propanol in an USC100T ultrasonic bath from VWR (Radnor, Pennsylvania) for 30 s, followed by a stream of dry nitrogen gas. The coverslip was then placed in the microscopy setup and heated to 200 °C. At this temperature, the polymer melted and was thus carefully spread over the coverslip and covered by a second coverslip to avoid contact with ambient humidity and achieve more even and comparable sample thicknesses of (10 ± 5) μm.

Observations using POM were conducted with isothermal temperature profiles. After the samples are molten at 200 °C, the system was allowed to equilibrate for 3 min. Melt as well as cold crystallization was observed. For melt crystallization, the sample was cooled to 140 or 130 °C respectively, without water cooling. For Cold crystallization, the samples were rapidly cooled to 35 °C, by the aid of the water-cooling setup. The system was again allowed to equilibrate for 3 min. Then temperature was enhanced to 65 °C. Evaluation of the recorded temperature profile showed an average cooling rate of (26 ± 1) K/min from 200 to 130 and 140 °C without water cooling, and (597 ± 29) K/min from 200 to 50 °C with water cooling. The heating rate of the system from room temperature to 65 °C was (127 ± 33) K/min. Samples were used for multiple temperature treatments.

Pictures were taken at regular intervals. Every 5 s for melt crystallization and every 3 min for cold crystallization at 65 °C. The camera settings were ISO 800 with an exposure time of 1/50 s for melt crystallization and ISO 3200 with an exposure time of 1/4 s for cold crystallization at 65 °C. The aperture was fixed by the camera mount on the microscope. Brightness and contrast of the images taken during cold crystallization are digitally optimized using Photoshop 2023 (Adobe, San Jose, California).

### 2.11 Atomic Force Microscopy

Atomic force microscopy (AFM) measurements were performed with a 5600LS High Resolution Large Stage AFM (Keysight Santa Rosa, California) in Acoustic Intermittent Contact Mode to measure the samples surface topography. As probe, a Tap300Al-G by BudgetSensors (Sofia, Bulgaria) with a beam shaped tip and a tip radius of 10 nm was applied.

The samples were prepared by melting the compounds on AFM metal specimen discs, 10 mm by Plano GmbH (Wetzlar, Germany) in the heating stage of the microscope and rapidly cooled by manually placing them on a sheet of lab grade aluminum foil. The samples stayed predominantly amorphous as proven by respective DSC measurements (data not shown). The temperature treatment was done in UF 110 Plus oven from Memmert (Schwabach, Germany) at 65 °C for 48 h. AFM scans were conducted with an edge length size of 30 μm × 30 μm, a resolution of 1024 lines and points per lines and later cropped to 25 μm × 25 μm.

## 3 Results and Discussion

### 3.1 Influences of Crystallization on the Direct Light Transmission during Temperature Treatment

The compounds were temperature treated as described in Section 2.3. As expected, samples tempered at higher temperatures showed a more rapid crystallization. When the average direct light transmission *T* reached a stable value in all compounds, temperature treatment was stopped, resulting in different temper durations. At one given temperature specimens of all materials were tempered for the same duration. The crystallization kinetics are discussed in more detail in Section 3.2.

In Figure 1 photographs of the samples on a support with a regular dot pattern (the dots are about 1 mm apart) are shown, taken before and after temperature treatment. Initially, no significant difference can be seen between the untreated samples, as the dotted background is clearly visible behind neat PLA and the PLA compounds. Regardless of the temperature, neat PLA samples turned completely and homogeneously opaque such that the background is not visible anymore, a result of strong scattering. PLAEBS samples show significantly reduced scattering after temperature treatment while the distribution of the haziness is slightly inhomogeneous. With decreasing tempering temperature, the clouding in the compounds significantly decreases until the background can be discerned behind the sample treated at 65 °C for 64 h (3840 min). The materials with EBHS appear the least turbid and after temperature treatment at 65 °C for 64 h, the dotted background is clearly visible although the sample appears slightly hazy.

**Figure 1:**
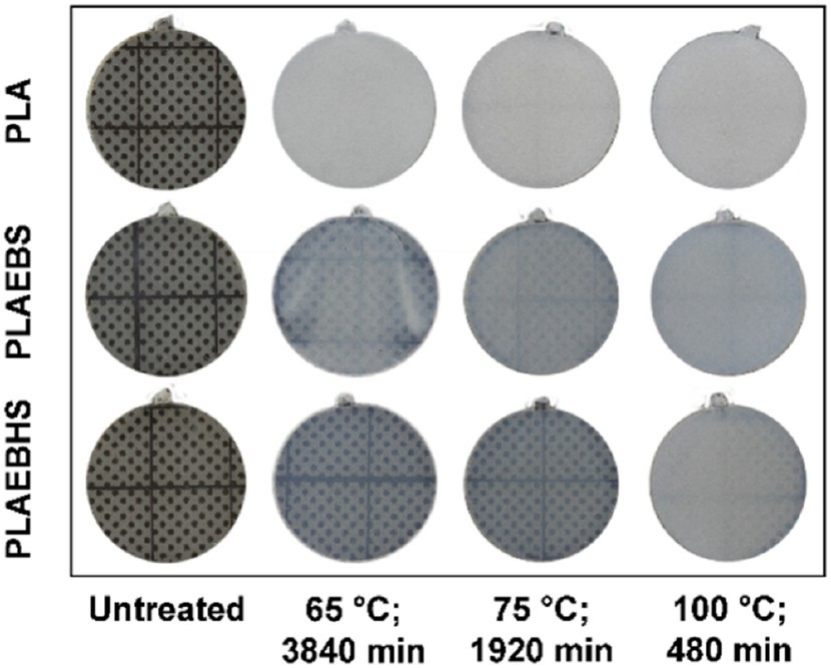
Photographs of PLA, PLAEBS and PLAEBHS samples before and after temperature treatment at different temperatures.

The direct transmission spectra of all samples are measured before, during and after temperature treatment and the averaged transmission per material and temperature is calculated by eq 1. In Figure 2a, the averaged transmission spectra *T*(λ) of the samples treated with 65 °C are given before (dashed line) and after temperature treatment (solid line). The spectra show the direct transmission to decrease the most in the shorter wavelengths’ region for all materials. This is typical for scattering. Transmission spectra of the samples treated at 75 and 100 °C are provided in the Supporting Information.

**Figure 2:**
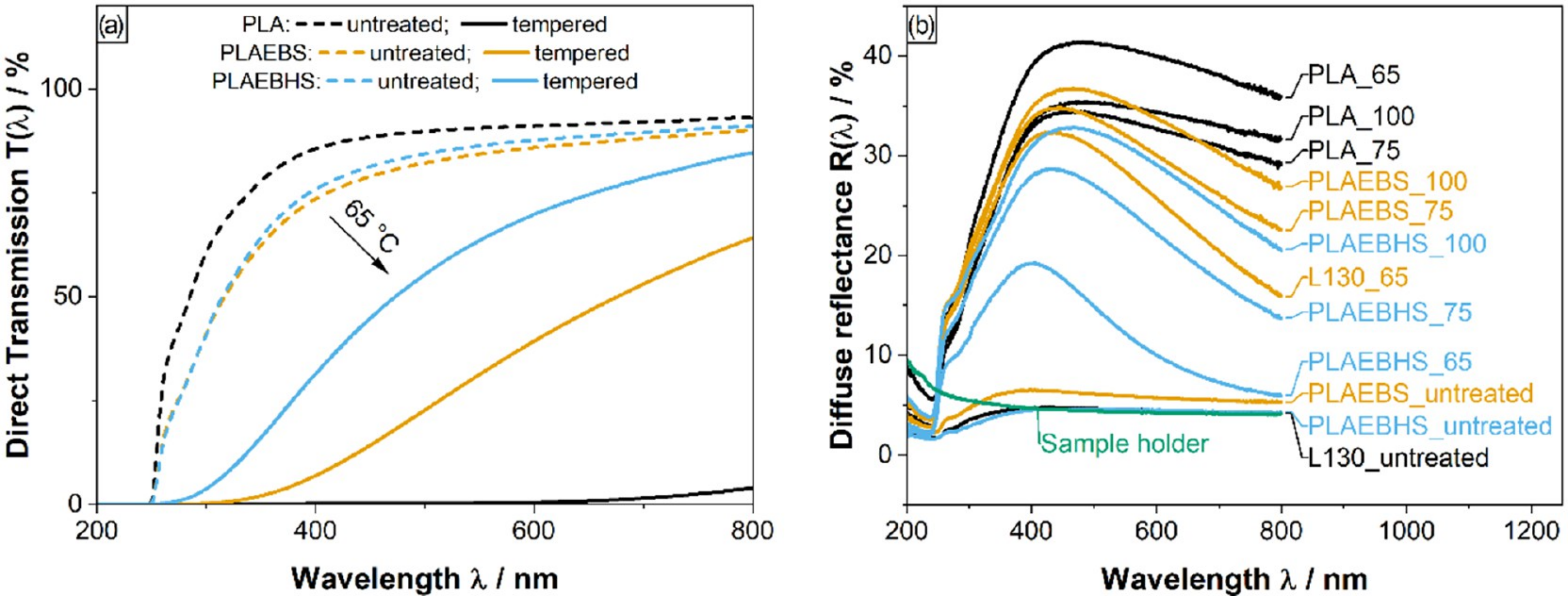
(a) Averaged direct transmission spectra *T*(λ) of PLA, PLAEBS and PLAEBHS samples before and after temperature treatment at 65 °C (five samples each). Dashed lines indicate the untreated samples. (b) averaged diffuse reflectance spectra of the samples measured subsequently to temperature treatment and aging at 80 °C. All averages are calculated in accordance to eq 1.

Further indication for Mie scattering is found in reflectance spectra measured on presumably semicrystalline samples (after temperature treatment), as shown in Figure 2b. For characterization, the samples were places in a black, absorbing sample holder to suppress background reflection. As some of the samples exhibit substantial transmission in the relevant wavelength region, the sample holder is expected to quantitatively influence the data, which is accounted for by providing a background spectrum of the holder. Untreated (presumably amorphous) samples show almost no diffuse reflectance, whereas thermally treated samples display significant reflectivity. Neat PLA shows the highest overall reflectance, while samples containing fatty acid amides show a reduced intensity of reflected light across the entire spectral range. Additionally, the reflectance spectra show a distinct wavelength maximum. Both the reduced amplitude and the shift of the maximum toward shorter wavelengths in additive-containing samples are consistent with Mie scattering theory, which predicts wavelength-dependent scattering by particles or domains on the order of the wavelength of light. The observed changes therefore suggest that the additives may lead to the formation of smaller or less refractive crystallites.

By the application of eq 2 the averaged direct transmission *T* in the visible was calculated from the measured spectra. The results are shown in Figure 3. These were obtained at the same time as the photographs (Figure 1). Initially, a direct transmission *T* of (90.4 ± 0.4) % for PLA, (83.8 ± 2.7) % for PLAEBS and (85.7 ± 1.5) % for PLAEBHS, were obtained. This shows that the additives slightly decrease the overall transmission for samples in the glassy state. This decrease could be due to the fact that the mixtures may not be fully homogeneous, and the blending of the polymer and additive is not yet optimal. During temperature treatment, the direct transmission *T* of neat PLA dropped to (0.8 ± 0.4) % (100 °C), (0.258 ± 0.009) % (75 °C), and (0.229 ± 0.002) % (65 °C). The average direct transmission *T* of PLAEBS increased with decreasing tempering temperature, ranging from (3.1 ± 3.2) % after tempering at 100 °C, to (11.5 ± 7.0) % after 75 °C temperature treatment and finally (34.9 ± 6.5) % after 3840 min at 65 °C. The highest transmission was measured for PLAEBHS compounds, starting from (10.6 ± 0.5) % after 100 °C treatment, (27.8 ± 10.0) % after tempering at 75 °C and finally (63.1 ± 2.9) % after 65 °C temperature treatment. These measurements confirm the visual observations in Figure 1.

**Figure 3:**
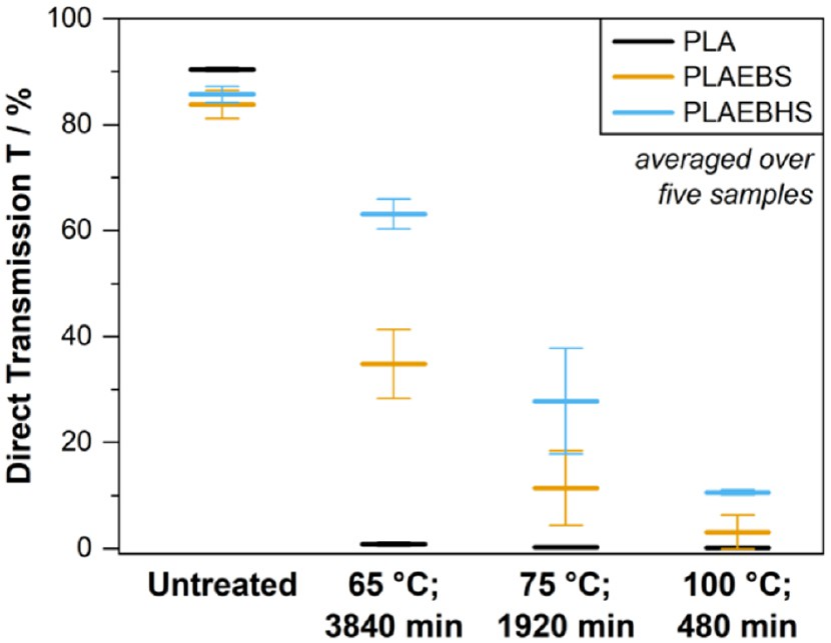
Average direct transmission *T* in the visible of PLA, PLAEBS and PLAEBHS (five samples each), before and after temperature treatment. *T* was calculated using eq 2.

Since indications for scattering were found in UV–Vis spectroscopy measurements, the progressions of the diminishing transmission during the temperature treatment seem to represent a reasonable reference to the crystallization speeds. For all materials, this progression is given at 65 °C in Figure 4. Progressions at 55 °C, 75 and 100 °C are provided in the Supporting Information. Since sampling time intervals are doubled after each measurement, the abscissa is plotted on a logarithmic scale. During temperature treatment at 65 °C, the first signs of clouding were detected after 60 min in the PLAEBHS samples. 60 min later, first signs of clouding appear in the PLAEBS samples. The pure PLA samples took about 240 min for clouding to set in. This indicates the onset time of the crystallization to double from PLAEBHS to PLAEBS and again to PLA. Nevertheless, given the considerable time intervals between individual measurements, the obtained values must be regarded as approximate.

**Figure 4:**
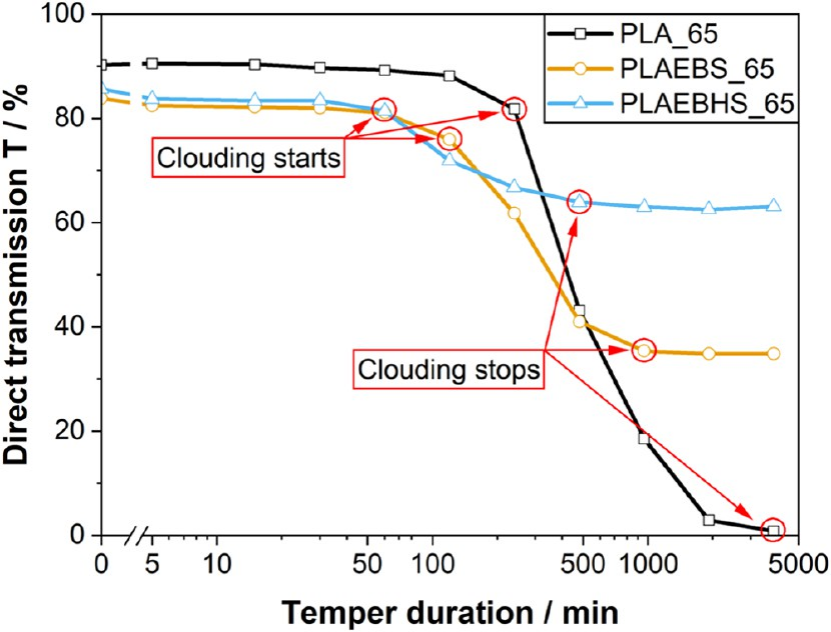
Changes in direct light transmissions *T* of PLA (black squares), PLEBS (yellow circles) and PLAEBHS (blue rectangles) samples during the temper process at 65 °C. The direct transmission *T* was calculated using eq 2.

An according behavior is also detected for the duration until clouding to stop. PLAEBHS stopped first after 480 min duration of tempering. Thereafter the clouding in PLEBS samples stopped after 960 min and finally, pure PLA stopped at 0% transmission after 3840 min at 65 °C. From these measurements the time needed to complete the crystallization can be estimated. PLAEBHS took 420 min to fully crystallize, while PLAEBS took 840 min and PLA took 3620 min, i. e. PLAEBS takes twice as long as PLAEBHS to fully crystallize while pure PLA takes 9 times as long as PLAEBHS.

The analysis of local molecular structures and dynamics can be performed by observing the longitudinal *T*_1_ relaxation time via temperature dependent ^1^H NMR measurements. Using the BPP theory, correlation times τ_c_ of the observed motional modes of the individual samples are determined and compared with each other.^41^

When looking at the *T*_1_ data in Figure 5a, a strongly pronounced relaxation mode in the range of 75 to 170 °C can be recognized for all samples. BPP fits of these modes (see SI Figures 10–12) result in correlation times in the range of 10^–6^ s, which is in the typical range of an α-relaxation process due to glass transition of the polymer segments. As can be seen in Figure 5a, addition of EBS or EBSH leads to a lower temperature onset of these motional modes and consequently to a lowering of the *T*_g_ of PLA, as expected for a plasticizer. The prerequisite for the crystallization process is a sufficiently large mobility of the chains, which is given at temperatures around and above *T*_g_ up to the melted state. At crystallization temperatures around *T*_g_, as carried out while measuring the direct light transmissions (see Figure 4), the *T*_1_ data therefore indicates a faster crystallization start (“clouding” in Figure 4) for PLAEBHS and PLAEBS in this order compared to the pure PLA, which is in excellent agreement with the results above. Looking at the correlation times of the α-relaxation mode resulting from BPP Fit (see Figure 5b), it can be determined that these are slightly reduced by the addition of additives. Addition of EBSH and EBS therefore ensures faster molecular movement in this regime.

**Figure 5:**
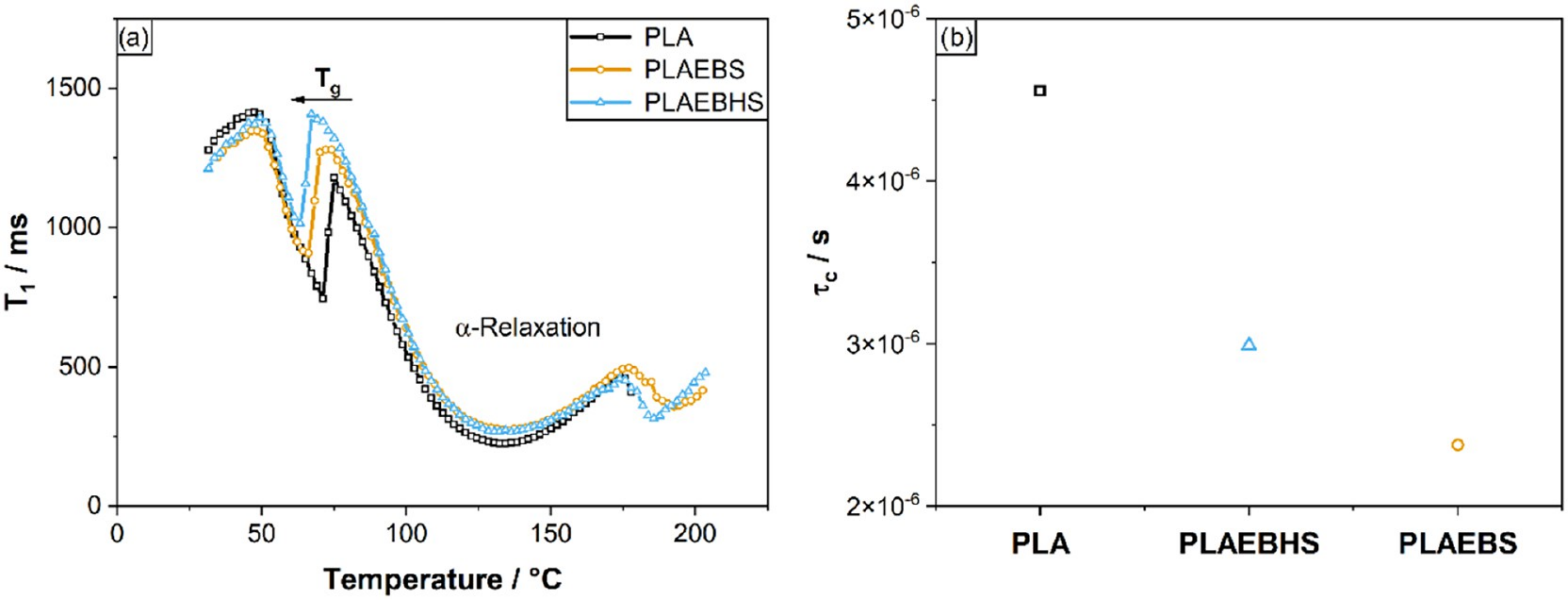
(a) Temperature dependent longitudinal relaxation rates (*T*_1_) for PLA (black) and mixtures of it with additives EBS (yellow) and EBHS (blue) measured at a Larmor frequency of 20 MHz. (b) Motional correlation time τ_*c*_ calculated at 298 K established via BPP fits of the α-relaxation modes from *T*_1_ data at 30–200 °C.

To test the hypothesis regarding the crystallization rates, DSC is a preferred method. Nonisothermal measurements were performed for the samples before and after temperature treatments. The respective thermograms are displayed in Figure 6. The untreated samples show a well-defined glass transition with *T*_g_ at 58 °C (PLA), 57 °C (PLAEBS) and 58 °C (PLAEBHS), determined at the inflection point. Furthermore, a cold crystallization peak can be identified with respective crystallization onset temperatures (*T*_c_) of 89 °C (PLA), 80 °C (PLAEBS) and 77 °C (PLAEBHS). As this temperature is highly dependent on the crystallization rate, the shift of approximately 9–12 °C indicates a nucleating effect of the additives.^48^

**Figure 6:**
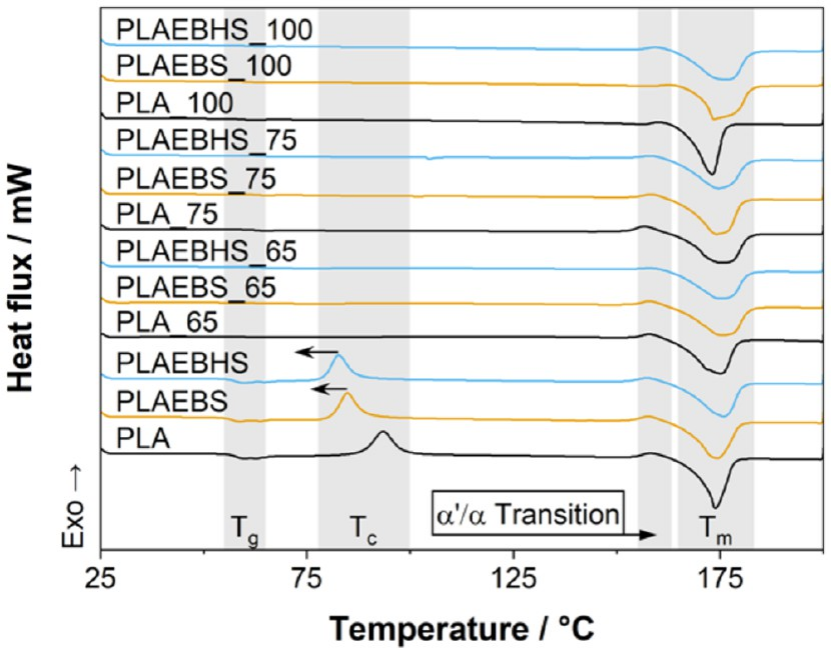
First heating DSC thermograms before and subsequent to thermal treatment at different temperatures.

At approximately 155 °C a smaller exothermal signal is detected. This behavior is well-known for PLA crystallized below 120 °C and has been subject of earlier studies. By correlating DSC measurements, with synchrotron X-ray-scattering, Kawai et al. found that the crystallization temperature greatly effects the crystal structure of PLA. At temperatures below 120 °C the polymer predominantly crystallizes in the less ordered and less stable α′-form. On heating recrystallization into the more stable α form at around 150 °C is observed, the temperature is depending on the given PLA grade. Kawai et al. also suggested that this is a solid-state phase transition, due to the comparably high crystallizations speeds. This process is often referred to as α′/α transition.^34,49^ 175 °C is the melting temperature (*T*_m_) of PLA resulting in a strong endothermic peak. Its broadening is attributed to the melting and recrystallization of different crystal forms, including the α′ and α-form.^50^

From the DSC data, the relative crystallinity of the materials can be calculated. As only the initial crystallinity is relevant, all crystallization enthalpies (including α′/α transition) were subtracted from the melting enthalpy. Therefore, a linear arbitrary baseline was defined from the first onset of crystallization, until the last trace of melting. The positive area above the baseline was accepted to represent Δ*H*_c_ and the negative enthalpy to be Δ*H*_m_. Crystallinity *X*_c_ was calculated using eq 4. Table 2 shows the calculated values. As melting and recrystallization overlap at temperatures between *T*_c_ and *T*_m_, there will be a comparably high uncertainty in these results. Furthermore, no correction was made considering the variation of specific heat capacities with temperature nor aggregate condition. However, the calculated crystallinities certainly render a valid approximation of *X*_c_ and prove all tempered samples to show a similar, relative crystallinity of 55–62%, which is in the typical range for fully crystallized PLA.^47^ The additives do not appear to influence the crystallinity of PLA.

**Table 2:** Crystallinities Calculated from the Thermograms in Figure 6 Using eq 4

	crystallinity *X*_**c**_			
sample	untreated (%)	65 °C; 3840 min (%)	75 °C; 1920 min (%)	100 °C; 480 min (%)
PLA	5	56	55	62
PLAEBS	2	55	60	57
PLAEBHS	6	58	57	57

Variable temperature (VT) and isothermal solid-state ^13^C­{^1^H} cross-polarization (CP)/MAS NMR spectroscopy experiments were carried out on pure PLA and PLAEBS to gain insight into the structure of the growing crystals upon heating. We utilize the magnetization transfer mechanism of the CP sequence, which is based on through-space heteronuclear dipole–dipole coupling to enhance the signal intensity and lower the signal-to-noise ratio while measuring ^13^C MAS NMR spectra.^51,52^ Figure 7a shows solid-state ^13^C­{^1^H} CP/MAS NMR spectra of pure PLA in the temperature range from 30 to 130 °C. At 30 °C there are three broad resonances with characteristic ^13^C NMR shifts at 170.0 ppm (COO(1)), 69.5 ppm (CH(2)) and 16.9 ppm (CH_3_(3))­of the pretempered fully amorphous sample, as expected. Upon heating near and above *T*_g_, splitting of the resonance signals of the carbonyl signal becomes discernible as shoulders at 98 °C. As investigated in detail by Pan et al., splitting of these signals refers to the existence of the α′ and α-crystal structure.^53,54^ Figure 7b shows isothermally recorded ^13^C­{^1^H} CP/MAS NMR spectra of PLAEBS at 98 °C in the time range of 0 and 12 h. Given that the same characteristic splitting of the ^13^C resonance lines is observed as in pure PLA, it can be inferred that the rapid formation of both α′ and α crystallites also occurs in the presence of the additive during heating.

**Figure 7:**
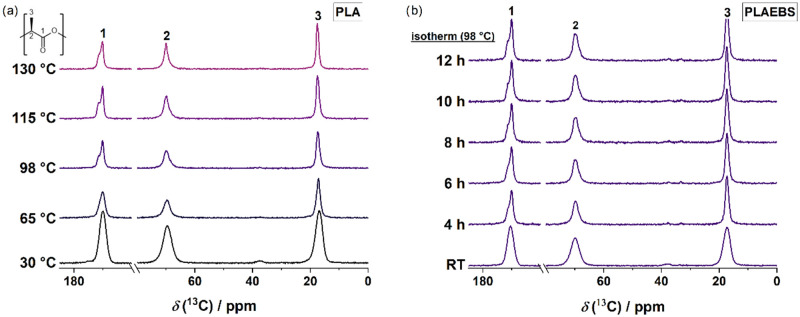
(a) Variable temperature ^13^C­{^1^H} CP/MAS NMR spectra of PLA. (b) Isothermal ^13^C­{^1^H} CP/MAS NMR spectra of PLAEBS recorded at isothermal conditions at 98 °C.

To get further insight into the formation of crystallites, ATR FTIR absorption was measured during temperature treatments. The results during the treatment at 65 °C are shown in Figure 8. At the beginning only one absorption band can be distinguished in the region between 1310 and 1290 cm^–1^ and none in the region close to 920 cm^–1^. Later measurements show an emerging second absorption band to be distinguished at around 1290 cm^–1^. Also, a band is emerging in the 920 cm^–1^ region. Both of these arising bands have been associated with PLA in its crystal phase. Furthermore, Praveena et al. could assign the two bands between 1310 and 1290 cm^–1^ to the α′-form, while the α form would lead to the absorption band to shift further apart in terms of wave numbers.^55^

**Figure 8:**
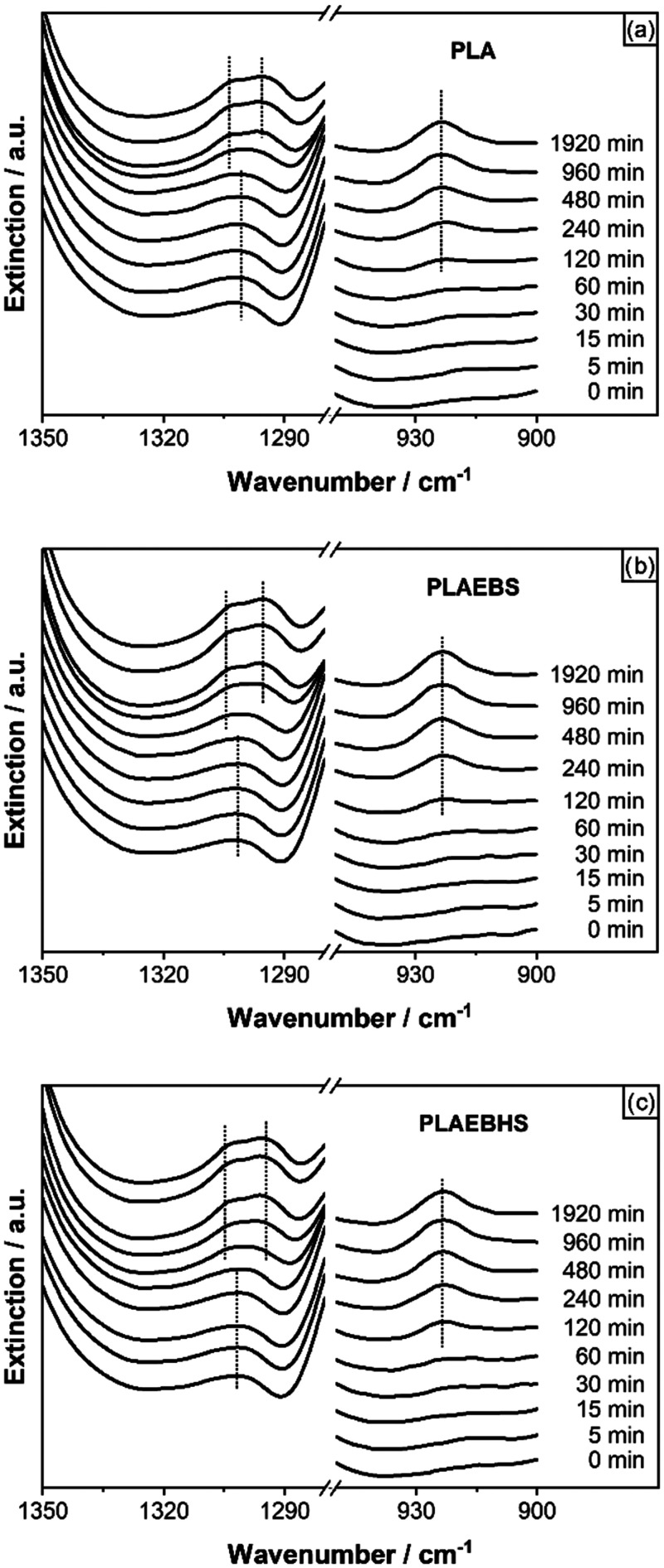
Ge ATR FTIR absorption spectra measured during the tempering process at 65 °C of (a) PLA, (b) PLEBS and (c) PLAEBHS. The emerging absorption bands are associated with PLA α′ crystallization in the literature.^55^

The shown ATR FTIR measurements also confirm the accelerated crystallization, whereafter PLAEBHS (Figure 8c) compounds crystallize fastest followed by PLAEBS (Figure 8b) compounds and pure PLA crystallizing slowest (Figure 8a) among the analyzed samples.

To get further insights into the crystallinity of the tempered samples, XRD measurements were conducted. Respective outcomes are depicted in Figure 9a. For better comparison, all diffractograms are normalized to the intensity of the strongest (110)/(200) reflection at 2θ = 16°. A slight shift (<0.5°) of the occurring reflections is found in comparison to literature data. This can be assigned to small height offsets of the samples (not plane-parallel sample geometry) as well as mechanical stress. Since the samples were injection molded the latter seems reasonable, while the plane parallelism of the sample geometry was not assessed. However, the resulting crystal structure can be assigned to the ordered α-form of PLA crystallization.^56^ At 24.2° a reflection can be determined which is characteristic for the α′-form of PLA crystals.^57^ As this reflection is not quite distinctly recognizable in Figure 9a, the dedicated reader is referred to the Supporting Information, in which the diffractograms are provided in more detail and less compressed in direction of the ordinate. Apart from the slight shift of the reflections, not much of a difference among the materials nor temper temperatures could be identified by XRD. Samples before temperature treatment returned a completely amorphous signal.

**Figure 9:**
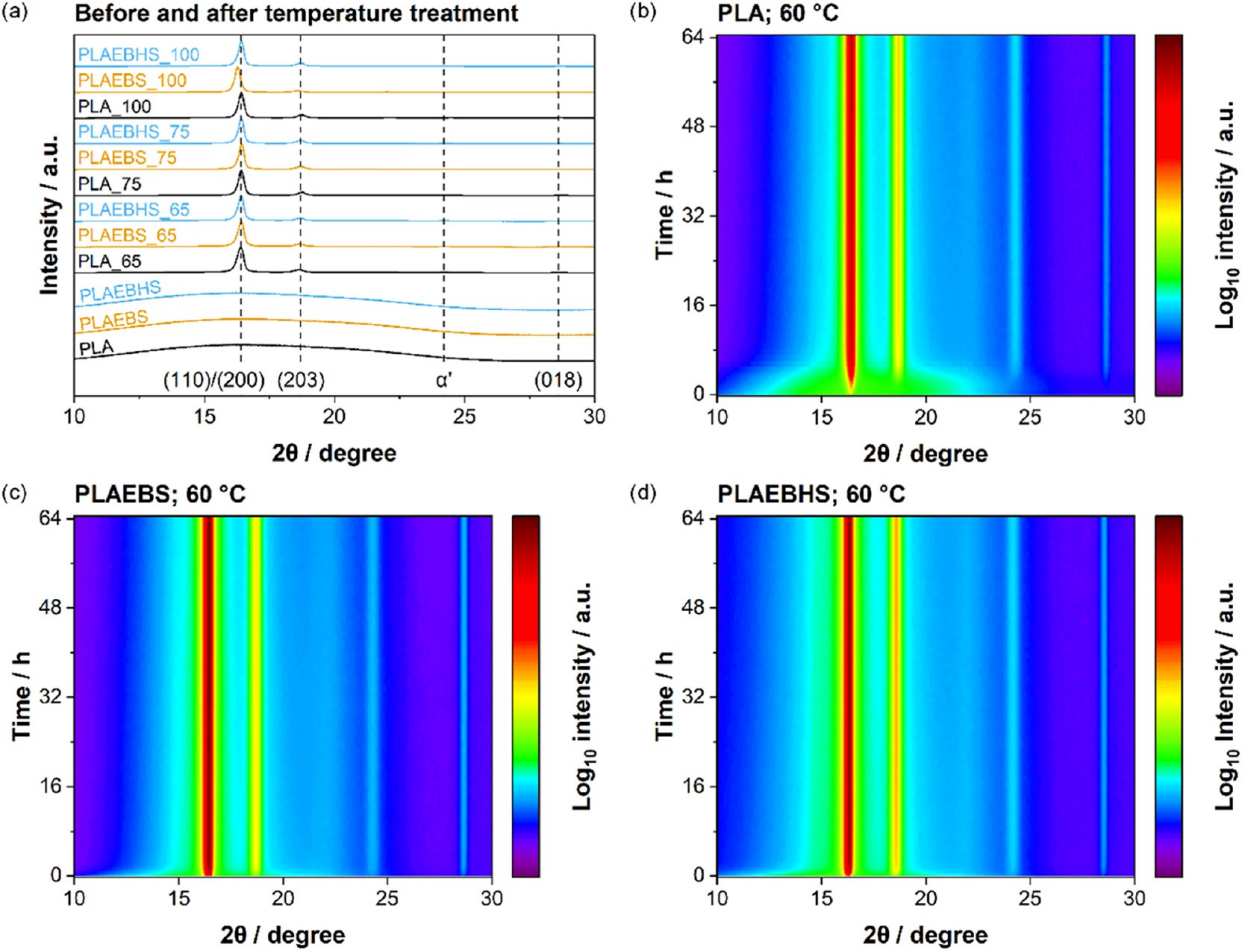
(a) XRD diffractograms of PLA, PLAEBS and PLAEBHS samples measured before and subsequent to thermal treatment at different temperatures, as indicated in the graph labels. Diffractograms of in situ temperature treatment with 60 °C are provided in (b) (PLA) (c) (PLAEBS) and (d) (PLAEBHS). All reflections can be assigned to the α′-form of PLA crystallization.

In situ temperature treatment at 60 °C was conducted in the XRD system. Time-resolved X-ray diffractograms are provided in Figure 9b,d with the rainbow colors representing the intensity and purple depicting zero while red represents the strongest signal. The initially amorphous signal of all samples is represented by a large green segment in the measurements at *t* = 0 h. Over time, the samples with additives show a significantly faster emergence of reflections, which is interpreted by the formation of crystallites. Already after 2 h of the in situ treatment, both PLAEBS and PLAEBHS return an almost completely semicrystalline signal. The neat PLA samples crystallized a lot more slowly, rendering a semicrystalline diffractogram only after 5 h. Since the intensity is plotted logarithmic, the reflections can be clearly identified. They all can be associated with the expected reflections for α′-PLA crystallites, as discussed above.

In comparison to the changes in light transmission measured with UV–Vis spectroscopy during the temperature treatment at 65 °C, the emergence of reflection appears earlier in the XRD experiments at 60 °C. Since both are linked to ongoing crystallizations and XRD was conducted at lower temperatures, these results seem contradictory. However, this behavior can be expected given that the samples had to be removed from the oven for UV–Vis spectroscopy and heat up every time they were placed back in the oven after the analysis had been performed. Depending on the heating time, this can result in the samples actually being at 65 °C for a shorter period of time. Furthermore, crystallites do not appear in UV–Vis spectroscopy until they are large enough to scatter visible electromagnetic radiation. Since XRD operates at a significantly smaller wavelength, crystallites are expected to be detected much earlier, possibly right after nucleation.

In summary the compound material samples maintained a strongly enhanced directed light transmission and were confirmed by different techniques to be in a semicrystalline state showing predominantly α′-crystals. Furthermore, the degree of crystallinity seems to be decoupled from the sample’s turbidity since the compound samples retain significantly higher direct transmission.

### 3.2 Crystallization Kinetics

Figure 10a shows a comparison of the crystallization kinetics of pure PLA, PLAEBHS and PLAEBS, resulting from evaluation of MSE-CPMG NMR experiments performed a *T* = 130 °C. While final degrees of crystallinity hardly differ, there is a clear difference in crystallization rates of the samples. The crystallization rate at *T*_cryst_ = 130 °C increased significantly with the addition of additives, indicating faster crystallization, particularly for the PLAEBHS sample. This is in good agreement with the DSC data (see Figure 6), as well as the direct light transmission measurements (see Figure 4).

**Figure 10:**
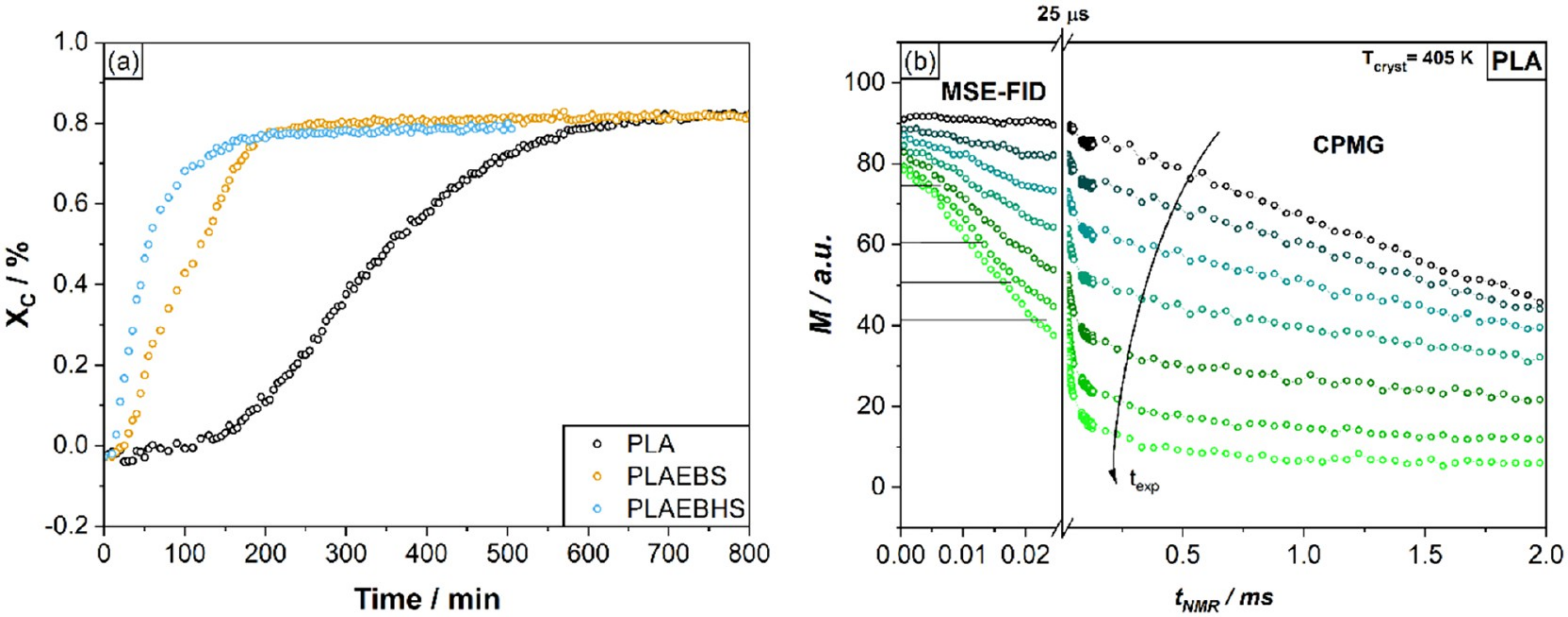
(a) Comparison of the isothermal crystallization kinetics of PLA (black), PLAEBS (orange), PLAEBHS (blue) as determined by MSE-CPMG NMR measurements. (b) Isothermal crystallization of PLA as monitored by MSE-CPMG NMR at 130 °C.

Isothermal melt crystallization experiments were conducted in a polarized light microscope equipped with a heating stage and observed in situ, see Figure 11. A time stamp is given on the bottom left corner of the micrographs, that includes cooling from 200 °C. Recorded temperature curves indicate the system to have cooled at a rate of (25 ± 0.6) °K/min, taking a total of 2:44 min – 2:51 min respectively. The scale bar indicates 250 μm. Figure 11a–c show pictures taken after about 4 min at 130 °C. In the micrograph of neat PLA small spherulites can be distinguished, while the samples with additives show significantly higher clouding, indicating a markedly increased number of crystals. Crystallization was considered complete when no more changes could be identified between multiple images. The images taken just when crystallization was considered complete are shown in Figure 11d–f. Considering the time stamp, crystallization in neat PLA appeared to have taken significantly longer in comparison to PLAEBS and PLAEBHS. Furthermore, the spherulites are noticeably larger in the neat PLA sample.

**Figure 11:**
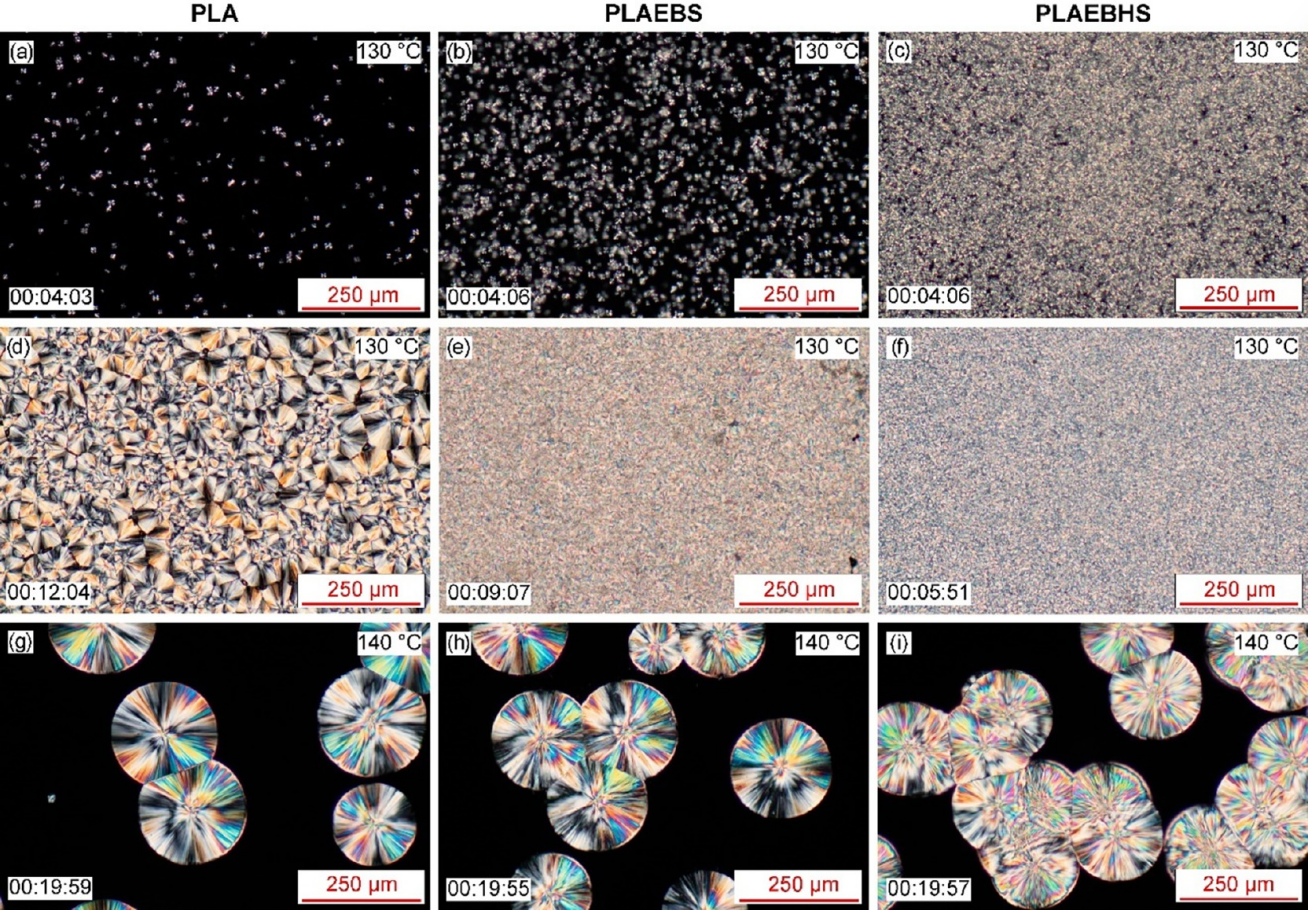
POM images taken of melt crystallized PLA, PLAEBS and PLAEBHS at 130 °C after about 4 min (a–c), when crystallization was complete (d–f) and at 140 °C (g–i). Timestamps are given on the bottom left, the scale bars indicate 250 μm.

When melt crystallization was induced at 140 °C (see Figure 11g–i), the effect of the additives was significantly reduced because these temperatures are very close to the crystallization point of EBS and EBHS. DSC analysis of other research groups show EBHS to crystallize around 134 °C during a cooling run with a 5 K/min cooling rate^27^ and 128 °C during a cooling rate of 30 K/min.^58^ In addition, the nucleation rate of PLA becomes very low. As a result, large crystals appear, reaching diameters of a few millimeters when allowed to grow. However, in Figure 11i, the number of crystallites in PLAEBHS blends still appears to be higher than in Figure 11f, for pure PLA.

The conducted POM investigations of in situ melt crystallization demonstrate that the effects of EBHS and EBS are most pronounced at temperatures below 140 °C. At a temperature of 130 °C, the effects are already substantial. When compared to the mechanisms widely accepted for PP clarifiers, the findings indicate that, segregation of EBHS in PLA appears between 130 and 140 °C. This is in agreement with the findings of Vo et al., who suggest this temperature to be between 180 and 100 °C.^58^

The temperature treatment at 65 °C was reproduced in POM to observe the crystallization processes in situ. Figure 12a,d,f show the samples after 13.5 h at 65 °C, this includes the duration for heating from room temperature to 65 °C. It can be seen that the pure PLA in Figure 12a shows no signs of crystallization, while PLAEBS (Figure 12d) and PLAEBHS (Figure 12f) crystallization is visible in form of small structural features – individual crystallites are not resolved. At this time the crystallization of PLABEHS appeared to be complete and no further changes in the sample could be detected in later images. After 17.5 h, crystallization was also complete in the PLAEBS samples (Figure 12e), while the pure PLA still showed no signs of crystallization (Figure 12b). Finally, after nearly 45 h, crystallization is complete in neat PLA (Figure 12c). All micrographs of the fully crystallized samples at 65 °C show a similar micro structure. Due to rotation of the plane of polarization in the crystallographic structures, the crystals appear brighter compared to amorphous regions. Upon close inspection, the microstructure in the PLAEBHS compounds appear more finely granular when compared to the neat PLA micrograph (please refer to the high-res image). This, in addition to the results on melt crystallization at 130 °C, supports the hypothesis of Chen et al. and Saitou et al. that EBHS reduce the crystal size in PLA.^27,29^ However, single crystallites can hardly be discerned as the resolution limit of the applied light microscopy setup is reached.

**Figure 12:**
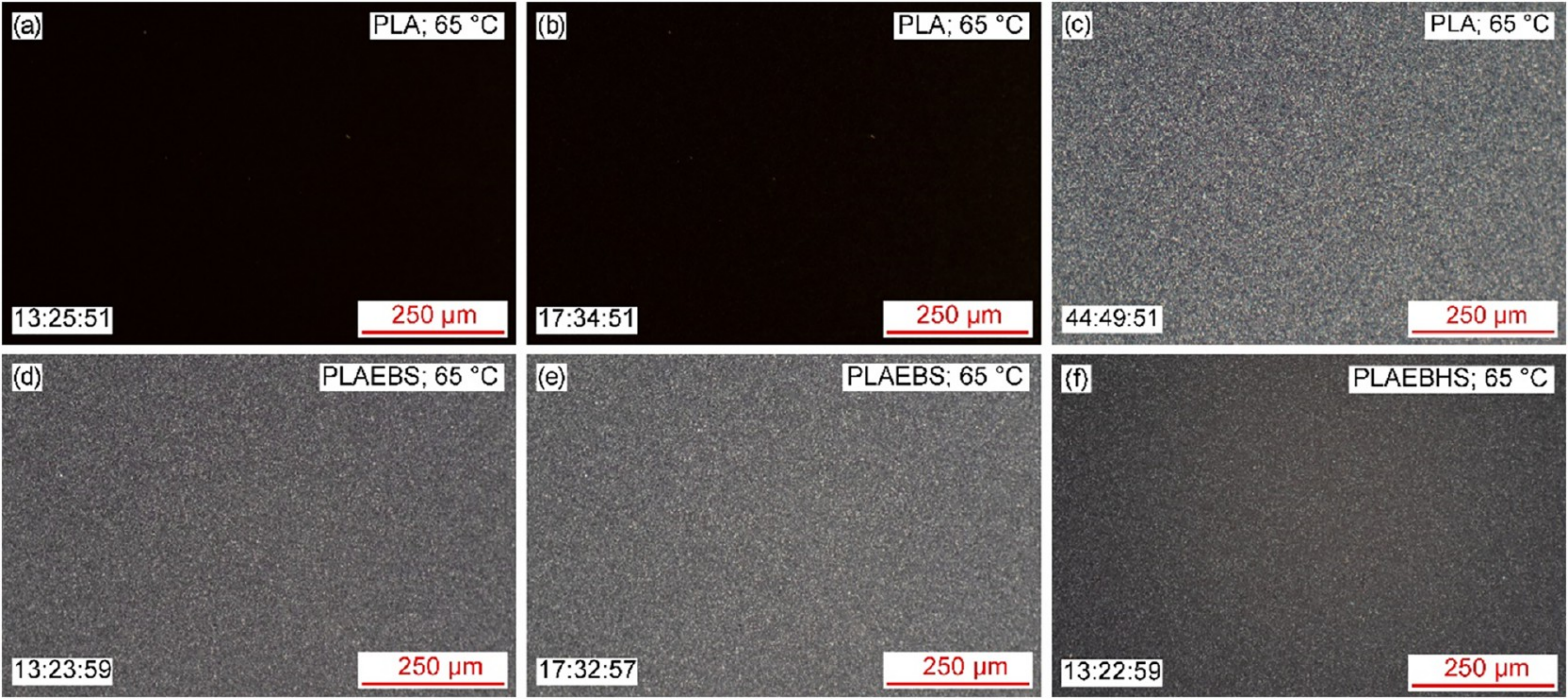
POM micrographs of cold crystallization at 65 °C in PLAEBHS (see f), in PLAEBS (see d, e) and in neat PLA (see a–c). A time stamp is given in the bottom left that includes heating from room temperature respectively. The scale bars indicate 250 μm.

In order to surpass the limited resolution of the POM setup, AFM measurements were conducted. These provide information on the sample surface topography with very high resolution (∼20 nm, according to the AFM-tip radius). Figure 13 shows topography scans of (a) quenched PLA, and of samples that have been temperature treated at 65 °C for 48 h (b–d). The results show a significant influence of the temperature treatment on the surface topography measured with AFM. The semicrystalline state of the temperature treated samples was confirmed by DSC measurements (data not shown). Furthermore, AFM measurements of crystals grown from melt crystallization at 130 °C (Supporting Information, and Figure 9) resemble the POM images of similar crystallites as shown in Figure 11a. It is therefore concluded, that the surface topography of PLA is significantly influenced by crystallization. Furthermore, the arising structures show the crystallite morphology to some extent.

**Figure 13:**
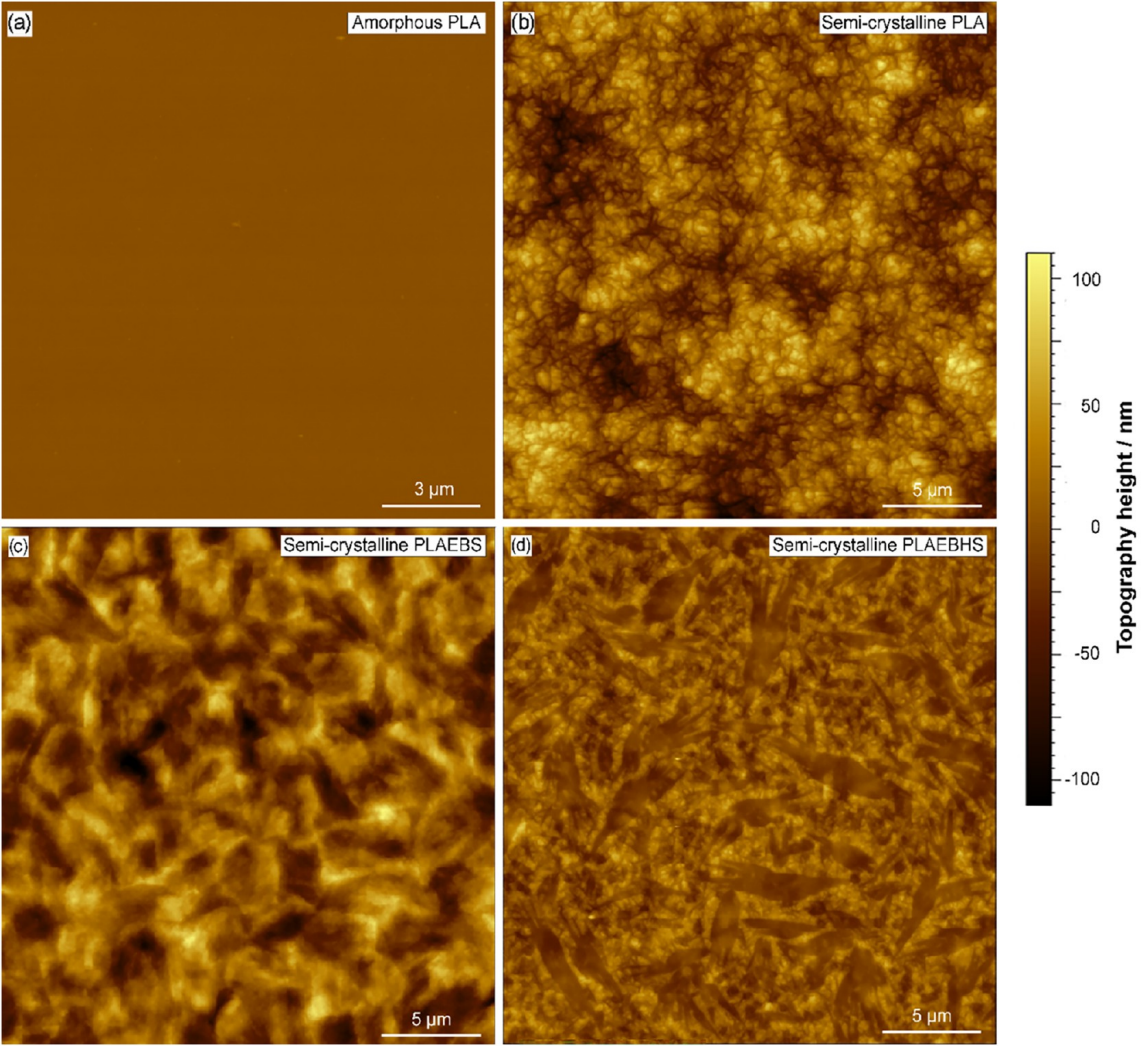
AFM topography images of (a) quenched PLA, and PLA (b) PLAEBS (c) and PLAEBHS (d) after temperature treatment with 65 °C for 48 h. The semicrystalline state of the compounds was confirmed by DSC (data not shown). The scale bars indicate 5 μm.

In Figure 13a, the quenched neat PLA sample exhibits an exceptionally plane surface with little to no structures. Subsequent to temperature treatment, neat PLA shows a granular texture with unevenly distributed clusters (Figure 13b). The topographical structure appears disordered, with small, irregular formations scattered across the surface. The height variations, indicated by the color gradient, suggest randomized growth and significant surface roughness. This roughness likely contributes to light scattering, resulting in hazing. In contrast, the PLAEBS sample (Figure 13c), reveals a more uniform and smoother texture, indicating that the additive promotes reduced surface roughness which suggests less light scattering, which could partially explain the observed reduction in haze.

PLAEBHS compounds (Figure 13d) show a surface with partially fibrillar structure, with elongated and well-organized pattern. The surface appears generally smoother, indicating that this additive induces pronounced structural effects. The reduced roughness and more ordered surface morphology likely add to the improved transparency seen with this additive.

### 3.3 Stability of Optical Properties at 80 °C

Finally, the stability of optical properties after temperature treatment was evaluated by exposing the samples to enhanced temperatures of 80 °C. Three samples of each material from all tempering experiments were placed in an aluminum tray and aged at 80 °C in an oven for 1 week. The direct transmission was measured afterward and is shown in Figure 14. While pure PLA remained completely opaque, the blends retained low haze. It is important to note that the direct transmission T of all the compounds tempered at 65 °C remained significantly higher during 1 week at 80 °C than the direct transmission T of the same materials tempered at 75 °C was before this aging experiment. A similar trend can be observed for all aged and tempered samples which retain significantly higher transmission compared to the unaged samples tempered at the next higher temperature.

**Figure 14:**
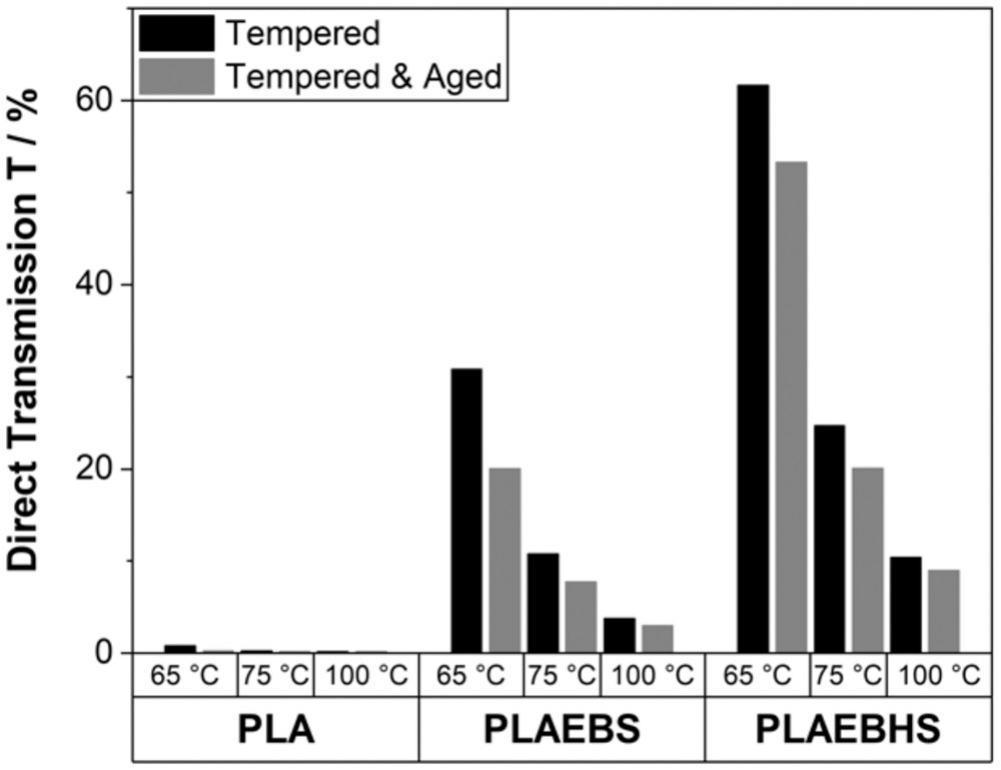
Direct transmission of all tempered materials before and after aging for 1 week at 80 °C. Note that the PLAEBS and PLAEBHS compounds treated at 65 °C retained higher direct transmission T compared to unaged samples after treatment at 75 °C.

## 4 Conclusions

The clouding of bulky PLA samples during crystallization was significantly reduced by the application of EBS and EBHS as additives in combination with low temperature treatment. We propose to introduce a dedicated tempering step in the material’s processing. Temperature has proven to be one major factor that strongly influences the spherulitic growth rate and nucleation time in PLA compounds. Generally, lower temperatures (below the maximum spherulitic growth rate) lead to shorter nucleation times as well as smaller radial growth rates of spherulites.^35^ Treatments at lower temperatures thus support the clarifying effect of EBS and EBHS. The results show the samples to exhibit stable visible light scattering properties even at elevated temperatures which surpass the respective crystallization temperatures.

While DSC and XRD measurements show the nucleating effect of EBHS and EBS in PLA, NMR analysis additionally suggests these additives to increase molecular motion in the polymer. This is also in agreement with the quicker albeit limited clouding observed during temperature treatments. XRD, FTIR and ^13^C­{^1^H} CP/MAS NMR all agree on the samples to contain predominantly the α′-crystal morphology. Furthermore, DSC also proofs the crystallinity to remain unchanged. MSE-CPMG NMR and POM investigations agree on a more rapid crystallization. The additives were proven to reduce the size of crystallite domains. AFM investigations proved the compound samples to exhibit lower surface roughness and a generally higher order in the surface structure. An increased light transmission caused by smaller crystallites with a higher order is in good agreement with the studies conducted by Stein et al.^20,21^

All in all, these studies show the feasibility to produce semicrystalline PLA with significantly increased optical transparency. Such a material will provide a biobased and biodegradable alternative for use as an optical polymer in lighting applications. Such environmentally beneficial materials are not available to this date.^59^

The study shows the clarifying properties of EBS and EBHS in PLA and complements the research with data for bulky samples. Additionally, dedicated NMR and AFM analytics were carried out for the first time and sophisticated low temperature tempering steps were investigated. We suggest to concentrate on EBHS over EBS as clarifier for PLA in optical use scenarios. Future work should also address fatty acid derivates with higher commercial availability in order to promote industrialization and leverage the promise of an environmentally beneficial alternative to PMMA or PC. Considering PLAEBS and PLAEBHS compounds, photothermal degradation should be investigated in order to assess and confirm suitable service life for lighting applications.

## Supplementary Material



## Notes

Transmission spectra recorded before and after thermal treatments at various temperatures, along with the corresponding changes in the averaged direct light transmission in the visible during the temperature treatment; X-ray diffractograms are presented in an enlarged format for improved clarity; additionally, an AFM scan of a sample area containing both amorphous and crystalline regions is included to illustrate their distinct morphological features; temperature-dependent longitudinal relaxation rates of PLA, PLAEBS, and PLAEBHS (PDF)
