# Supplementary material for: Toward Optical Quality Polylactide Using Fatty Acid Amides as Clarifiers
Source: Biomacromolecules. 2025 Aug 1;26(9):6177–91. doi: 10.1021/acs.biomac.5c01123 (PMC12421679; doi:10.1021/acs.biomac.5c01123)
Supplement: Supplementary file 1 [file bm5c01123_si_001.pdf]

Towards Optical Quality Polylactide using Fatty Acid Amides as Clarifiers

Supporting information

Kesting, Matthias Balthasar[a,b]*; Hochstädt, Sebastian*[c]*; Terbrack, Eric*[a]*; Ruder, Anna Maria*[a]*; Ahrens, Bernd*[d,e]*; Chassé, Walter*[c]*; Thomas, Christian*[a]*; Schweizer, Stefan*[d,e]*; Hansen, Michael Ryan*[c]*; Meyer, Jörg*[a,*]*; Seide, Gunnar*[b]

a Department Lippstadt 1, Hamm-Lippstadt University of Applied Sciences, Marker-Allee 76-78, 59063 Hamm, Germany

b Aachen-Maastricht Institute for Biobased Materials (AMIBM), Maastricht University, Bright-lands Chemelot Campus, Urmonderbaan 22, 6167 RD Geleen, The Netherlands

c Institute of Physical Chemistry, University of Münster, Corrensstraße 28/30, 48149 Münster, Germany

d Faculty of Electrical Engineering, South Westphalia University of Applied Sciences, Lübecker Ring 2, 59494 Soest, Germany

e Fraunhofer Application Center for Inorganic Phosphors, Branch Lab of Fraunhofer Institute for Microstructure of Materials and Systems IMWS, Lübecker Ring 2, 59494 Soest, Germany

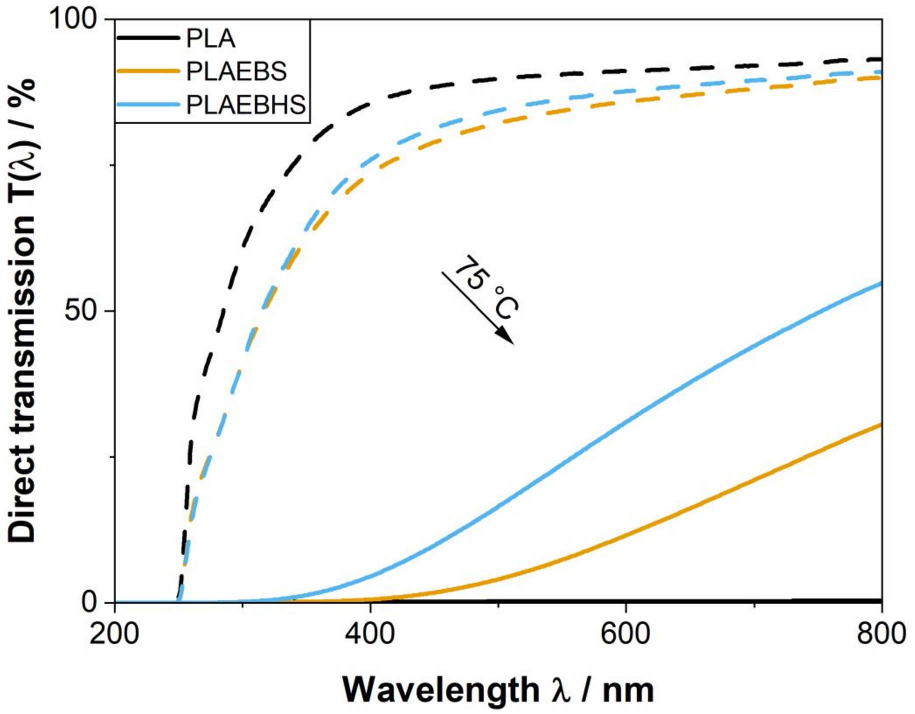

Figure 1. Averaged direct transmission spectra T(λ) of PLA, PLAEBS and PLAEBHS samples before and after temperature treatment at 75 °C (five samples each). Dashed lines indicate the untreated samples. Averages are calculated using equation (1).

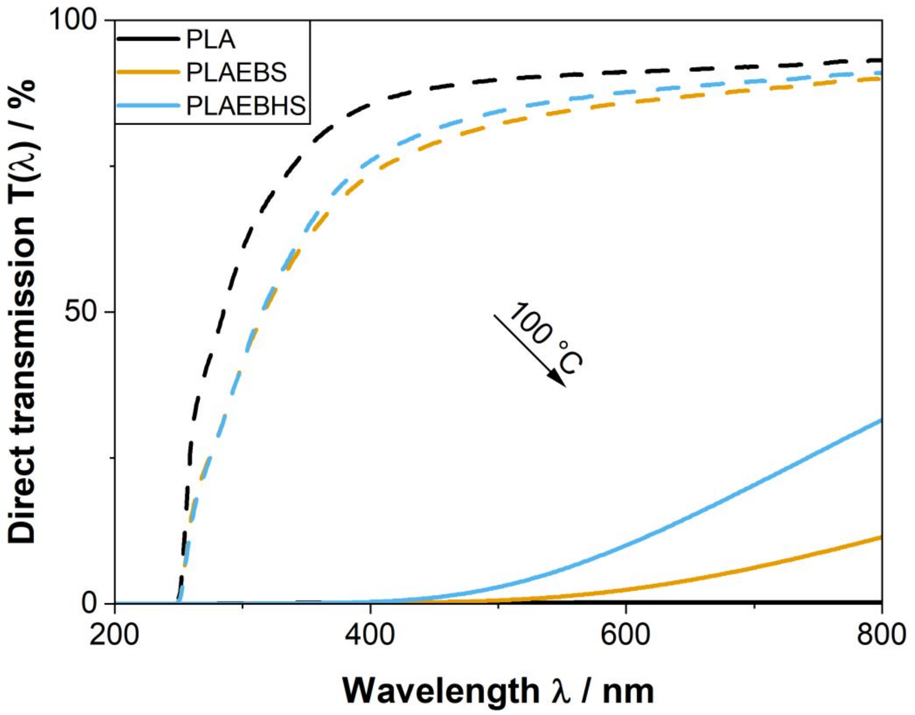

Figure 2. Averaged direct transmission spectra T(λ) of PLA, PLAEBS and PLAEBHS samples before and after temperature treatment at 100 °C (five samples each). Dashed lines indicate the untreated samples. Averages are calculated using equation (1).

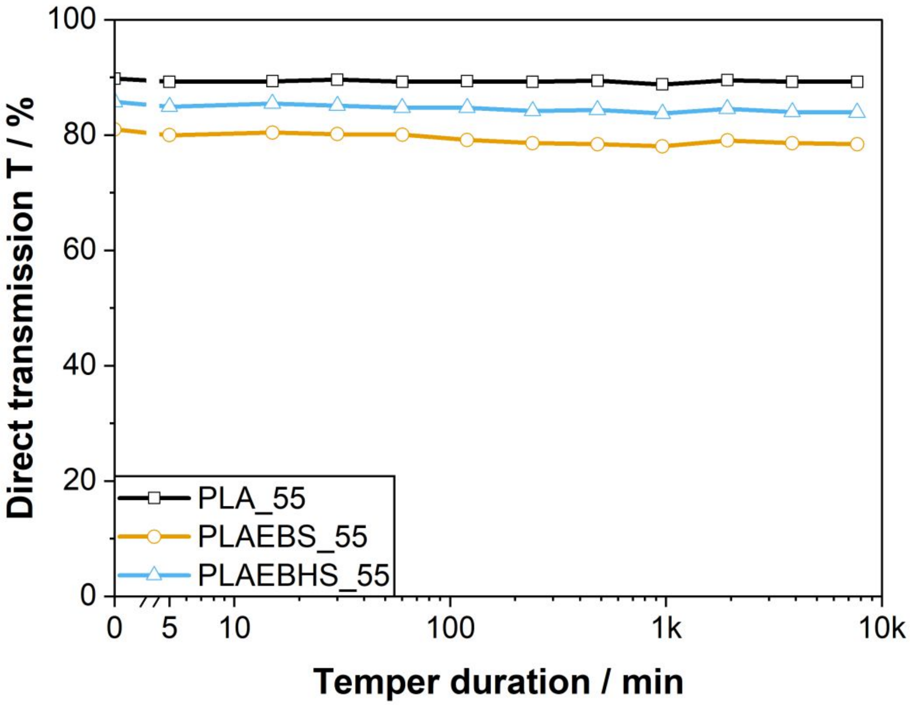

Figure 3. Changes in direct light transmissions T of PLA (black squares), PLEBS (yellow circles) and PLAEBHS (blue rectangles) samples during the temper process at 55 °C. The direct transmission T was calculated using (2).

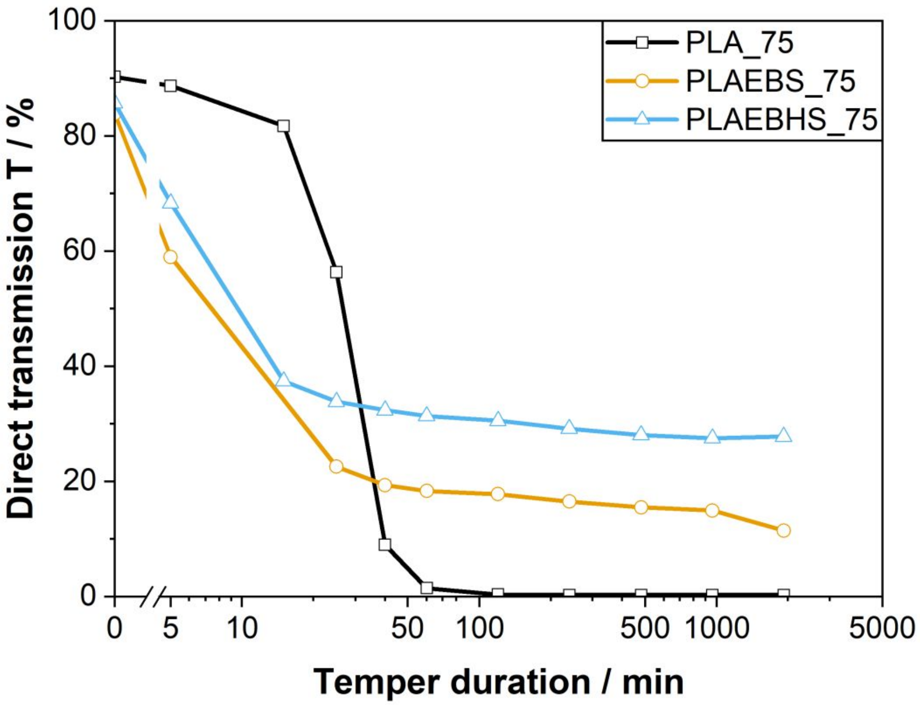

Figure 4. Changes in direct light transmissions T of PLA (black squares), PLEBS (yellow circles) and PLAEBHS (blue rectangles) samples during the temper process at 75 °C. The direct transmission T was calculated using (2).

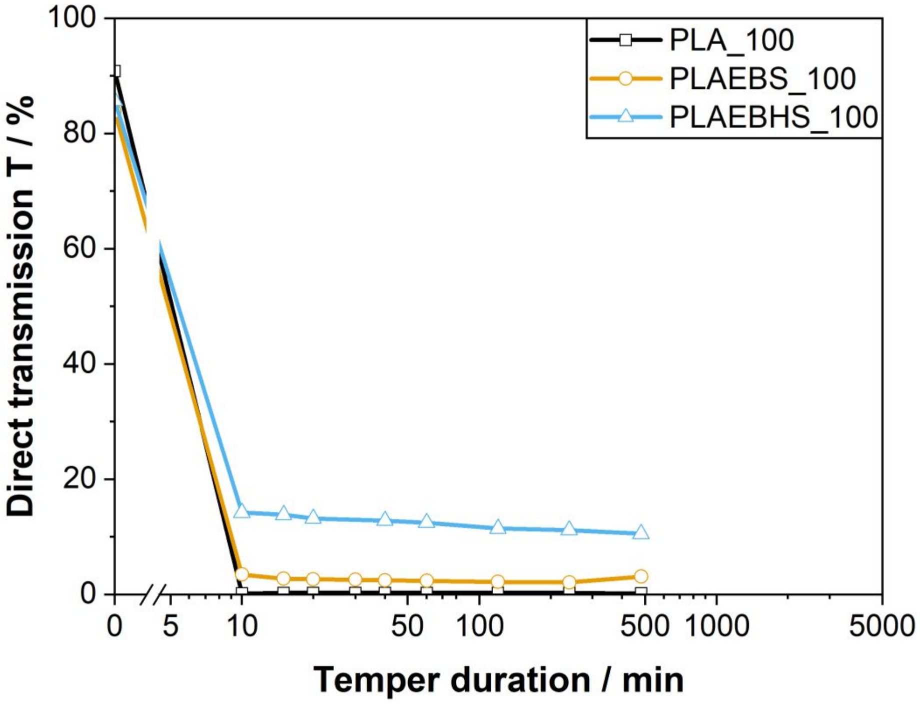

Figure 5. Changes in direct light transmissions T of PLA (black squares), PLEBS (yellow circles) and PLAEBHS (blue rectangles) samples during the temper process at 100 °C. The direct transmission T was calculated using (2)).

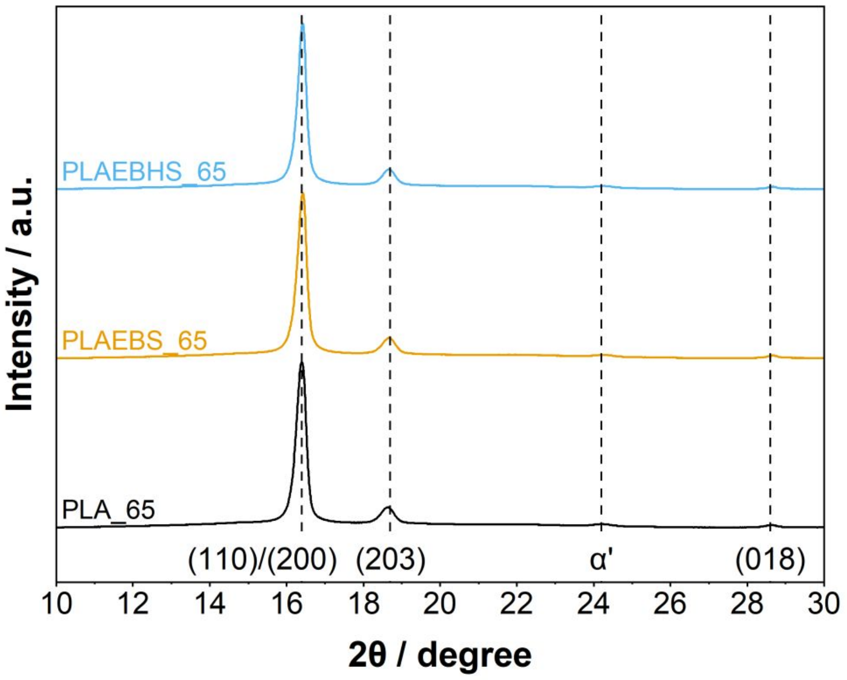

Figure 6. XRD diffractograms of PLA, PLAEBS and PLAEBHS samples measured subsequent to thermal treatment at 65 °C.

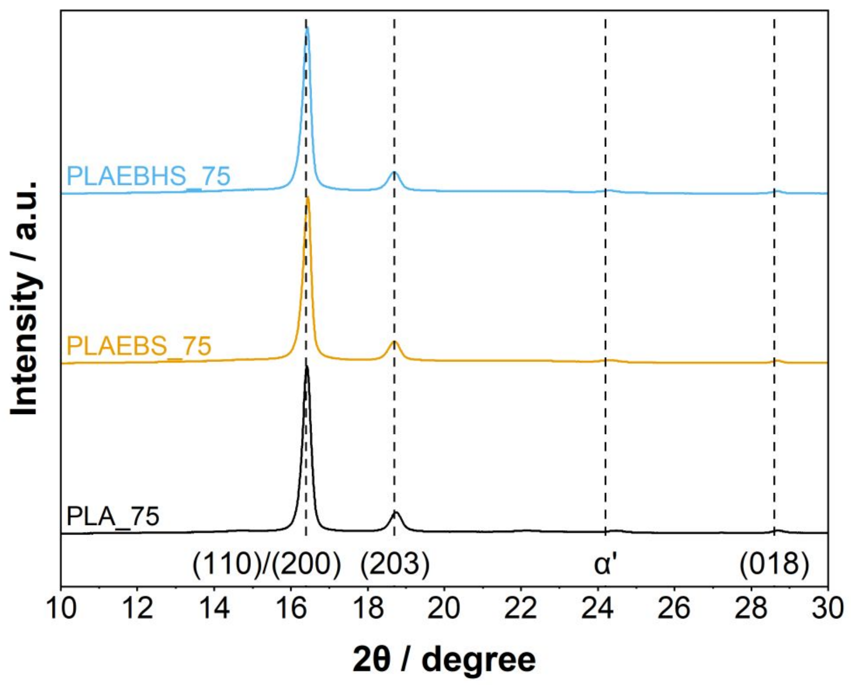

Figure 7. XRD diffractograms of PLA, PLAEBS and PLAEBHS samples measured subsequent to thermal treatment at 75 °C.

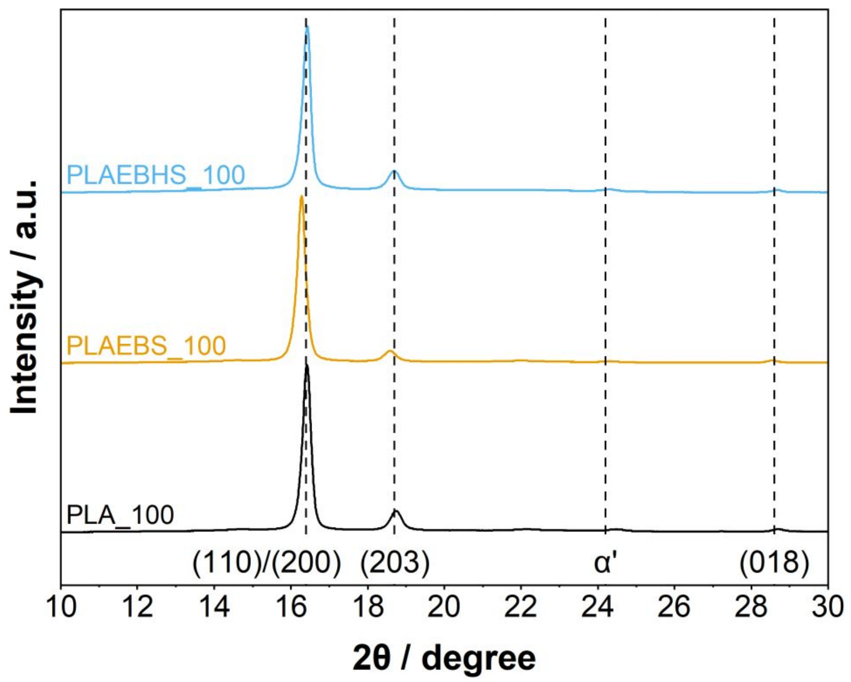

Figure 8. XRD diffractograms of PLA, PLAEBS and PLAEBHS samples measured subsequent to thermal treatment at 100 °C.

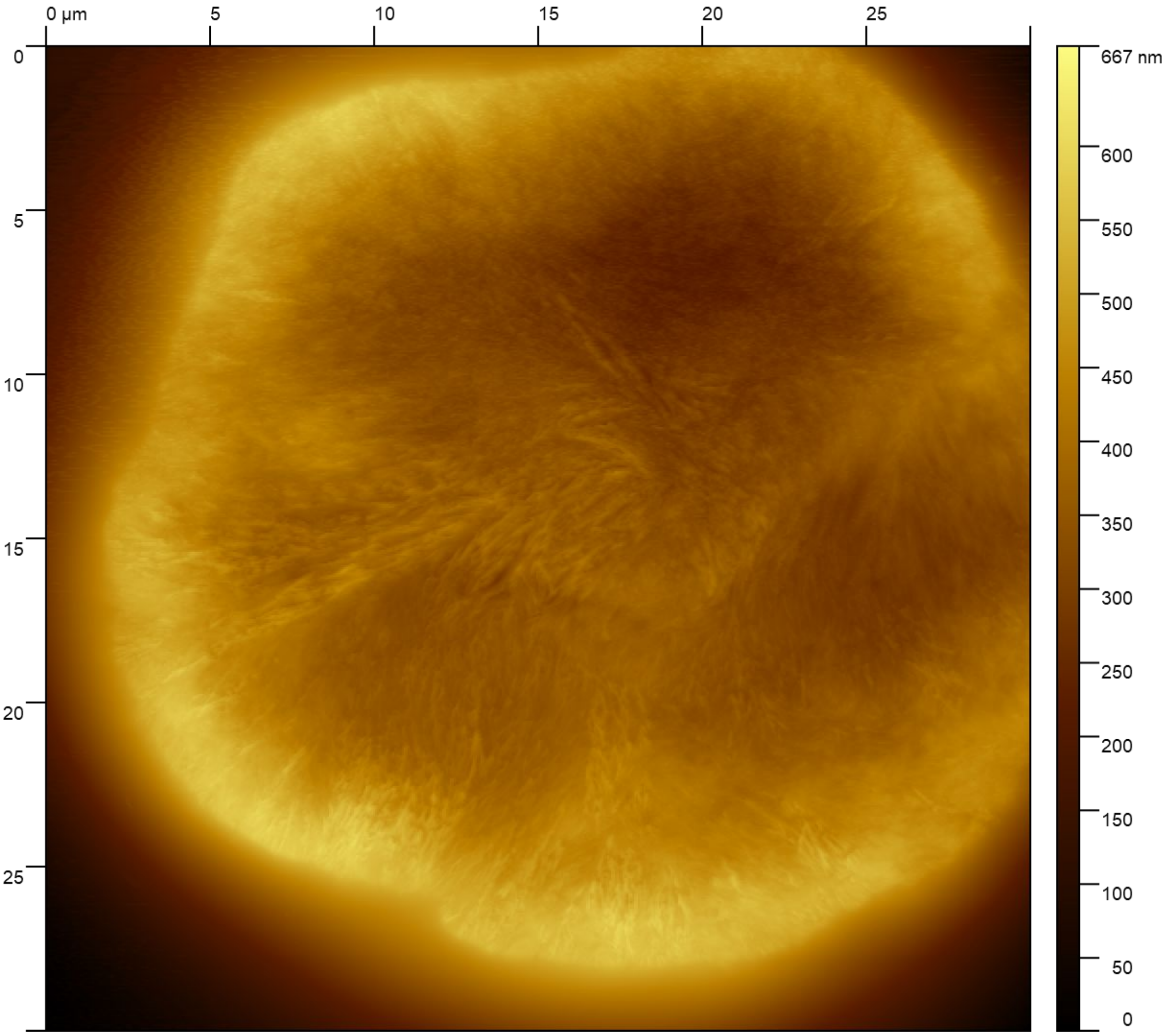

Figure 9. Topography of a PLA spherulite, grown at 130 °C and measured by AFM as described in the experimental section. The amorphous region around the crystal is distinguishable an appears significantly more flat.

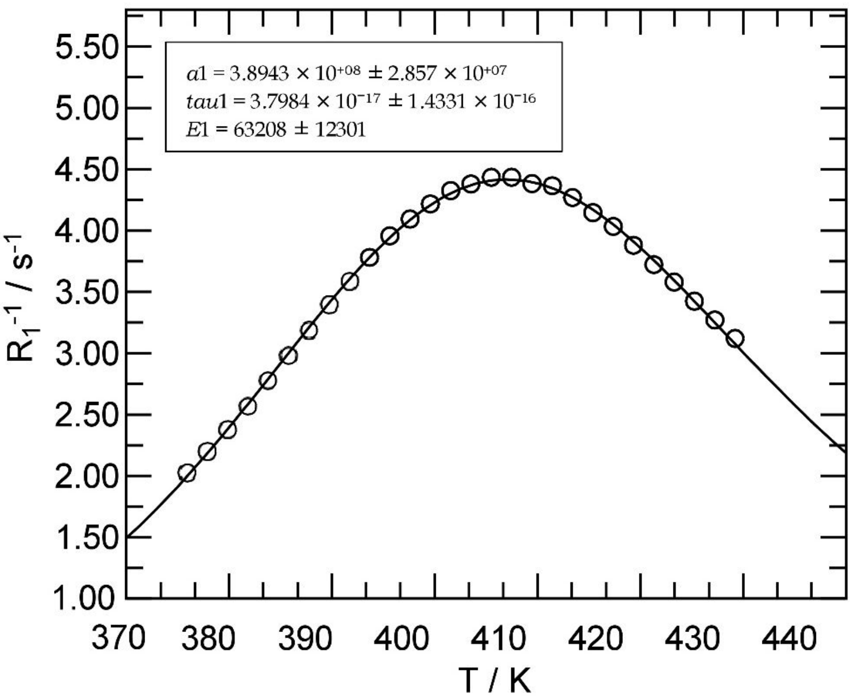

Figure 10. Temperature-dependent longitudinal relaxation rates (R_1) of PLA with representation of the best fit assuming a single BPP model.

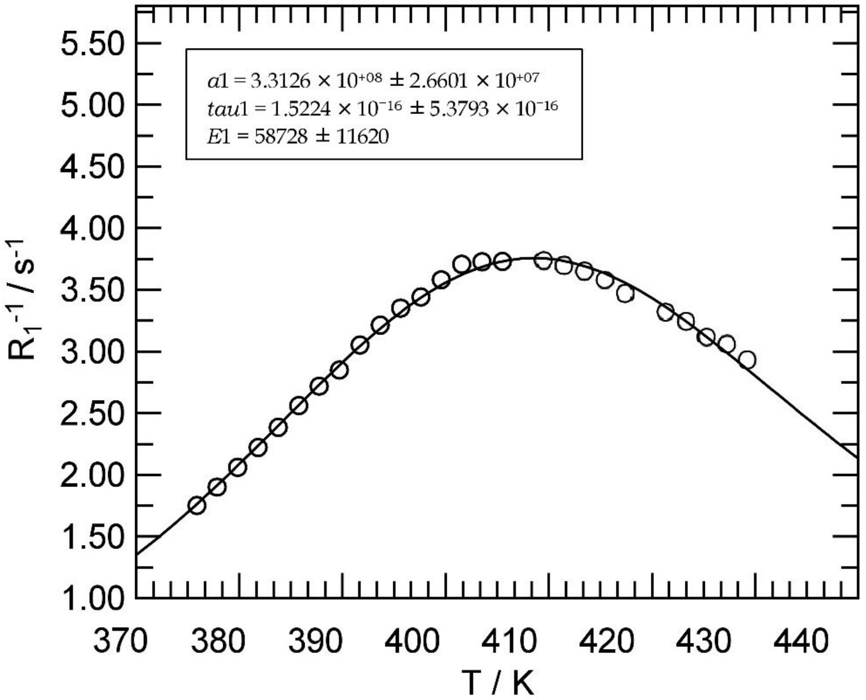

Figure 11. Temperature-dependent longitudinal relaxation rates (R_1) of PLAEBHS with representation of the best fit assuming a single BPP model.

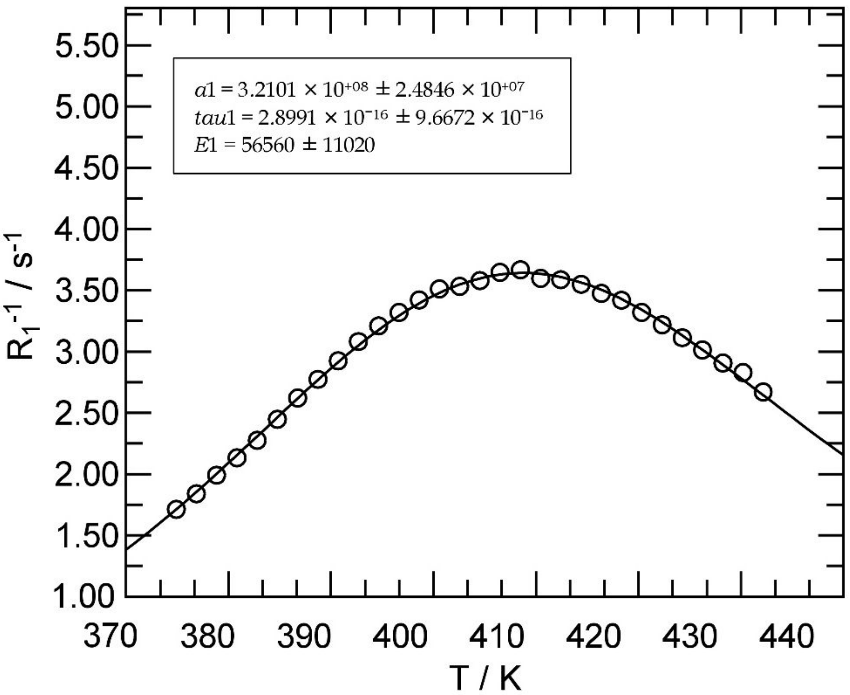

Figure 12. Temperature-dependent longitudinal relaxation rates (R_1) of PLAEBS with representation of the best fit assuming a single BPP model.
